# Identification of HOTf-Driven Brønsted Acid Catalysis in the AuCl_3_/AgOTf System for the Hydroalkylation of Styrene

**DOI:** 10.1021/acs.inorgchem.6c01693

**Published:** 2026-05-05

**Authors:** Amir Mahdian, Tatsiana Nikonovich, Sandra Kaabel, Kari Laasonen, Kaveh Farshadfar

**Affiliations:** † Department of Chemistry and Material Science, School of chemical Engineering, 174277 Aalto University, 02150 Espoo, Finland

## Abstract

The AuCl_3_/AgOTf catalytic system is widely used in C–C bond-forming transformations, yet the identity of its true catalytic species remains under debate. In this study, we examine whether the hydroalkylation of styrenes with 1,3-diketones, originally reported by Yao and Li, proceeds through a Brønsted acid pathway arising from the in situ generation of HOTf. Density functional theory (DFT) calculations show that formation of HOTf from Au­(OTf)_3_ is thermodynamically favored and kinetically accessible, accompanied by reduction of Au­(III) to Au­(I). These findings support a HOTf-mediated pathway over a gold-centered mechanism. The proposed mechanism involves the protonation of styrene, followed by the nucleophilic attack of the enol tautomer of the diketone and regeneration of HOTf via proton transfer to OTf^–^. Competing styrene dimerization is also predicted and experimentally observed as a side reaction. Comparative computational and experimental evaluation using other strong Brønsted acids supports the hypothesis that Brønsted acidity is the dominant driving force in this transformation. Taken together, these results indicate that, for the AuCl_3_/AgOTf system investigated here, the catalytically competent species is most plausibly an in situ generated Brønsted acid rather than a discrete precious-metal-based electrophile.

## Graphical abstract

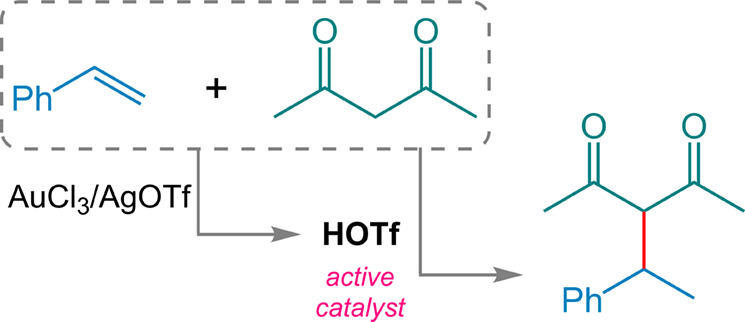

## Introduction

In recent years, gold has emerged as a versatile transition-metal catalyst with reactivity patterns distinct from those of more conventional late transition metals such as palladium, nickel, and copper. This distinctive reactivity has prompted extensive studies of gold in homogeneous catalysis and a wide range of organic transformations.^1,2^ Gold complexes exhibit pronounced carbophilic Lewis acidity, enabling efficient coordination to and activation of π-systems such as alkynes, alkenes, and allenes, thereby facilitating a diverse range of electrophilic transformations.^3,4^

The AuCl_3_/AgOTf catalytic system has been applied across various transformations, including Pictet–Spengler-type annulations,^5^ sulfoxide-activated glycosylation reactions,^6^ and gold-catalyzed cyclizations of allenic esters.^7^ These reactions typically proceed under mild conditions and are commonly rationalized in terms of highly electrophilic Au­(OTf)_3_ species generated in situ, enabling efficient activation of π-systems and high tolerance toward functional groups. At the same time, the behavior of Au/Ag catalytic systems can be highly dependent on the specific reaction manifold, the nature of the counterion, and the surrounding ion-pairing environment. Accordingly, conclusions regarding the identity of the catalytically competent species in one system should not be assumed to be generally applicable to other gold-catalyzed transformations.^8^ More broadly, weakly coordinating counterions in gold catalysis are not always merely latent sources of Brønsted acidity. In many systems, the counterion can remain an integral component of the catalytic assembly by influencing ion pairing and the reactivity of key intermediates.^8–10^

Among the earliest applications of the AuCl_3_/AgOTf catalytic system, Yao and Li developed an efficient intermolecular hydroalkylation of styrenes with 1,3-dicarbonyl compounds, demonstrating the system’s capability to promote C–C bond formation under mild conditions (upper part of Scheme 1a).^11^ Ariafard and co-workers^12^ conducted a DFT-based mechanistic study on the Yao and Li reported hydroalkylation of styrenes with 1,3-diketones. Based on their proposed mechanism, the reaction proceeds via the in situ formation of a benzyl cation promoted by Au­(OTf)_3_, which they identified as the actual catalytic species responsible for this transformation (Scheme 1a). Farshadfar and co-workers (2022) demonstrated that in the AuCl_3_/AgOTf-catalyzed annulation of phenol with cyclohexadiene, triflic acid (HOTf) is generated in situ through the irreversible reduction of Au­(III) to Au­(I). Their mechanistic analysis suggested that HOTf originates from the reaction of Au­(OTf)_3_, which is transiently formed in the catalytic system, with the substrates. DFT calculations confirmed that HOTf, rather than the gold complex, serves as the actual Brønsted acid catalyst promoting the annulation (Scheme 1b). Additionally, control experiments catalyzed solely by HOTf afforded the same product as the AuCl_3_/AgOTf system. In separate reactions catalyzed by AuCl_3_/AgOTf, Au­(I) and HOTf were detected.^13^

**Scheme 1:**
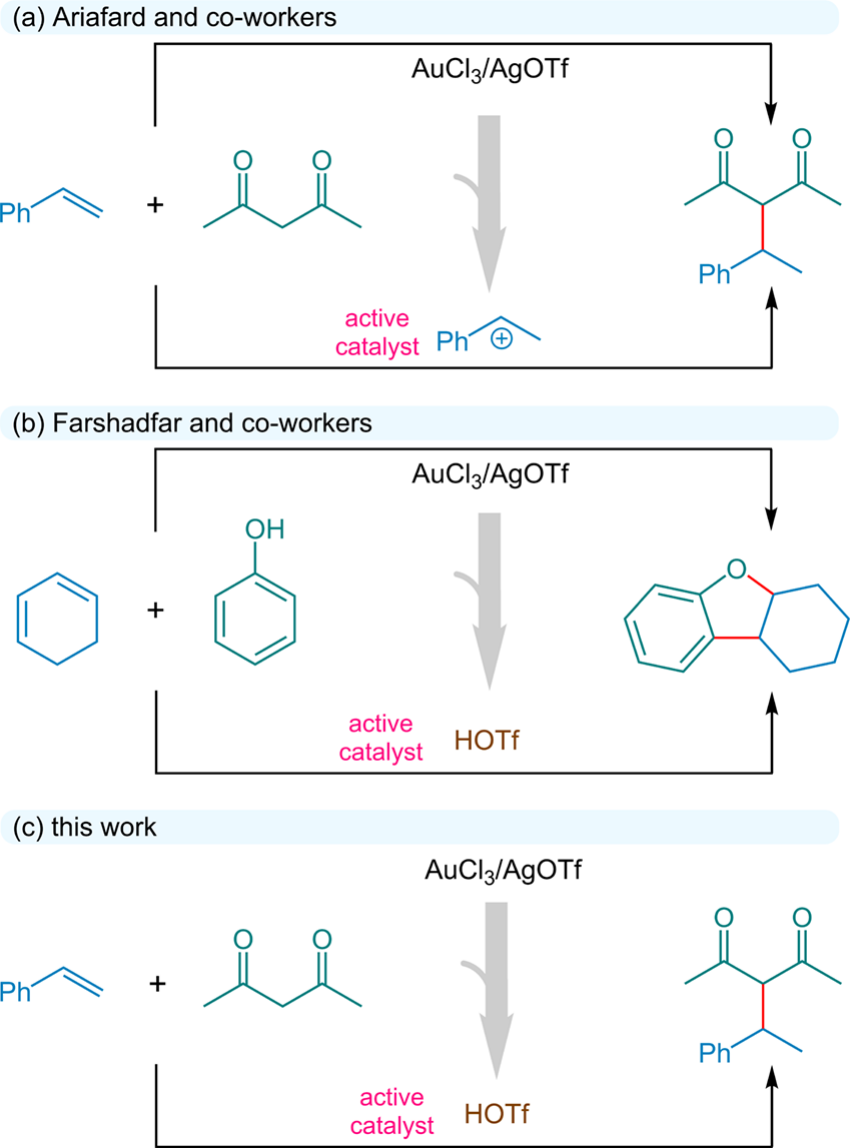
(a) Gold-Catalyzed Hydroalkylation of Styrene with Acetylacetone Using the AuCl_3_/AgOTf Catalytic System and the Proposed Benzyl Cation as the Active Species. (b) Proposed Reactive Intermediate in the AuCl_3_/AgOTf- or HOTf-catalyzed Annulation of Phenol with Cyclohexadiene. (c) Proposed Reactive Species Relevant to HOTf-catalyzed Coupling of Styrene with Acetylacetone

Motivated by the reduction-induced Brønsted acid formation mechanism proposed by Farshadfar et al., we hypothesize that HOTf can likewise be generated in situ within the AuCl_3_/AgOTf catalytic system employed by Yao and Li.^11^ To examine this possibility, we compare the Au­(OTf)_3_-mediated pathway proposed by Ariafard and co-workers^12^ with an alternative HOTf-based mechanism. We then validate this mechanism experimentally to assess its feasibility. Therefore, this study aims to address several key mechanistic questions: (i) Can triflic acid (HOTf) be generated in situ under the reaction conditions, similar to its formation in the Au­(OTf)_3_-catalyzed annulation of phenol and cyclohexa-1,3-diene?^13^ (ii) If HOTf is indeed formed under the reaction conditions, does it participate as an on-cycle catalytic species in the hydroalkylation process, or does it remain an off-cycle intermediate? (iii) How does this acid-catalyzed pathway compare kinetically with the mechanism proposed by Ariafard and co-workers? and (iv) If HOTf can promote the reaction in the absence of the gold precatalyst, could other strong Brønsted acids play a similar role, and can these hypotheses be validated experimentally?

## Results and Discussion

In this study, we investigated the mechanism of the AuCl_3_/AgOTf-catalyzed hydroalkylation of styrenes with 1,3-diketones originally reported by Yao and Li^11^ (Scheme 2a) using DFT calculations. As outlined in the Introduction, Ariafard and co-workers demonstrated that Au­(OTf)_3_ cannot serve as the genuine catalyst for this transformation. Instead, according to their proposed mechanism (Scheme 2b), coordination of the enol tautomer of the 1,3-diketone (**1** ^t^) to Au­(OTf)_3_ renders the hydroxyl proton highly acidic, allowing styrene (**2**) to readily deprotonate it. Consequently, protonated styrene forms a benzylic cation (**6**), which acts as the actual catalytic species responsible for this transformation. In this cycle, the benzyl cation (**6**) reacts with the enol form **1** ^t^ to form the C–C bond, giving the cationic intermediate (**7**), which is then deprotonated by another molecule of styrene to yield the final product (**3**) while regenerating the active catalyst (**6**).

**Scheme 2:**
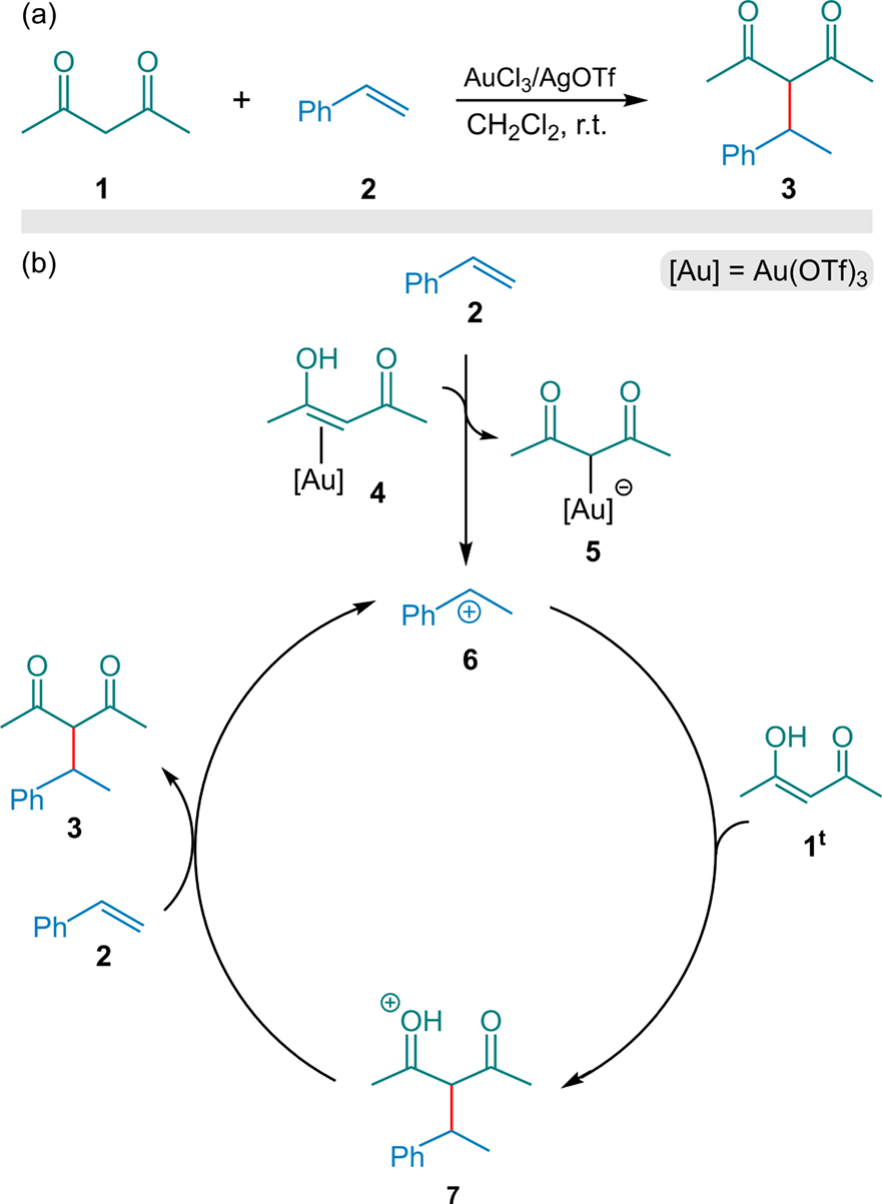
(a) Experimental Transformation Reported by Yao and Li. (b) Au­(OTf)_3_-based Mechanism Proposed by Ariafard and Co-workers Introduces Benzylic Cation **6** as the Active Catalyst

At this juncture, we investigated the potential formation of HOTf under the reaction conditions. Figure 1a compares the relative stabilities of various LAu­(OTf)_3_ complexes. Among these, species **8**, in which styrene coordinates to Au­(OTf)_3_, is found to be the most stable. In addition, our calculations indicate that diketone **1** can reversibly tautomerize to **1** ^t^ with a small energy cost of only 0.6 kcal·mol^–1^ (Figure 1b).

**Figure 1:**
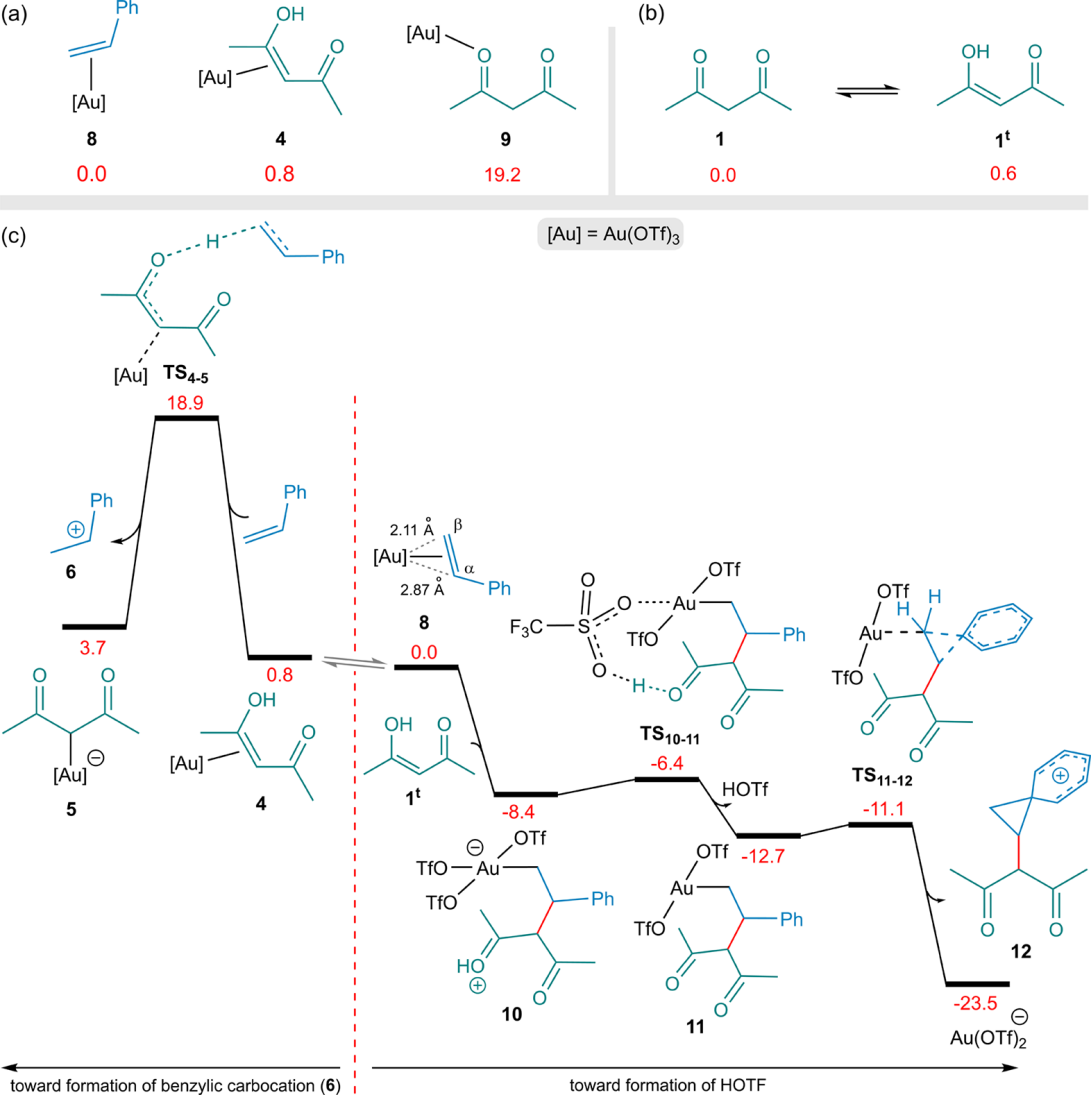
(a) Relative stabilities of LAu­(OTf)_3_ complexes. (b) Reversible tautomerization of diketone **1** to its enol form **1** ^t^. (c) Comparison of the computed free-energy profiles for the formation of the benzylic carbocation **6** (left) and the formation of HOTf (right). The relative free energies are given in kcal·mol^–1^ where CH_2_Cl_2_ is employed as the solvent.

In complex **8**, coordination to the Au­(III) center induces pronounced polarization of the styrene π-bond, leading to an elongated Au–C_α_ bond (2.87 Å) and a shorter Au–C_β_ bond (2.11 Å). The elongated Au–C_α_ bond is attributed to the development of significant carbocationic character at the C_α_ position, which is stabilized by conjugation with the phenyl ring. This enhanced polarization of the styrene π-bond arises from the strong electrophilicity of Au­(III), in conjunction with the weakly basic nature of the triflate ligands, which collectively increase the electron deficiency at the gold center. It is noteworthy that coordination bonds involving third-row transition metals are generally stronger than those formed by their lighter congeners with the same oxidation state and charge.^14^

The tautomer **1** ^t^ undergoes essentially barrierless C–C bond formation at the C_α_ center of complex **8**, as predicted at the employed level of DFT theory, yielding intermediate **10** at a relative energy of −8.4 kcal mol^–1^ (Figure 1c). The absence of an activation barrier can be rationalized by the markedly enhanced electron deficiency at the C_α_ center induced by Au coordination, together with the high nucleophilicity of **1** ^t^ arising from its electron-rich enol π-system, thereby facilitating a facile nucleophilic attack. Similar readily occurring C–C bond-forming processes involving gold-activated electrophilic centers have been previously reported.^13,15^

In intermediate **10**, the triflate ligand positioned trans to the carbon atom is most weakly bound to gold due to the strong trans influence of the carbon ligand, and therefore acts as the most basic among the triflates. Meanwhile, the proton attached to oxygen is highly acidic. Consequently, proton transfer from oxygen to the triflate ligand occurs through a low-energy transition state (**TS**_**10–11**_), affording complex **11** and releasing HOTf in an exothermic process (Δ*G*_*r*×*n*_ = −12.7 kcal·mol^–1^). Our calculations indicate that intermediate **11** can readily undergo reduction of Au­(III) via a transition structure **TS**_**11–12**_ with an S_N_2-type character to form Au­(I) in the form of Au­(OTf)_2_^–^. This process is accompanied by the formation of a phenonium ion **12** and is significantly exergonic. The resulting species **12** may further undergo conversion to more stable intermediates; however, these transformations fall outside the scope of the present discussion. As a result, all Au­(OTf)_3_ catalytic species ultimately convert into HOTf along with minor side products.

The left side of Figure 1c depicts the pathway leading to the formation of the benzylic carbocation **6**, previously proposed by Ariafard and co-workers as the actual catalytic species responsible for this transformation. Our DFT results, however, indicate that the formation of HOTf is markedly more favorable than the alternative pathway leading to **6**, which features a substantially higher activation barrier of 18.9 kcal·mol^–1^.

At this point, the key question arises: can HOTf itself catalyze the hydroalkylation reaction? To facilitate comparison and clarity in the following computational discussion, we set the reference of relative free energies to HOTf at 0.0 kcal·mol^–1^. According to our calculations, the Markovnikov addition of HOTf to styrene proceeds via the transition structure **TS**_**2–13**_, with an activation barrier of 14.1 kcal mol^–1^, and is slightly exergonic, with a relative free energy of – 0.8 kcal mol^–1^, leading to intermediate **13** (Figure 2a). Subsequent nucleophilic attack of the enol form of the diketone (**1** ^t^) on the carbon bearing the triflate moiety occurs via an S_N_2-type transition state (**TS**_**13–16**_) accompanied by triflate dissociation (Figure 2b). This step requires a relative free energy of 16.0 kcal mol^–1^. The liberated triflate anion can remain associated with the resulting cation as an ion pair. As OTf^–^ approaches the protonated oxygen (C=OH^+^), proton transfer occurs, regenerating the triflic acid catalyst HOTf and affording the final product **3**. Our calculations further show that the putative transition state for this proton transfer (**TS**_**16–3**_) does not correspond to a stationary point on the potential energy surface, consistent with a barrierless process.

**Figure 2:**
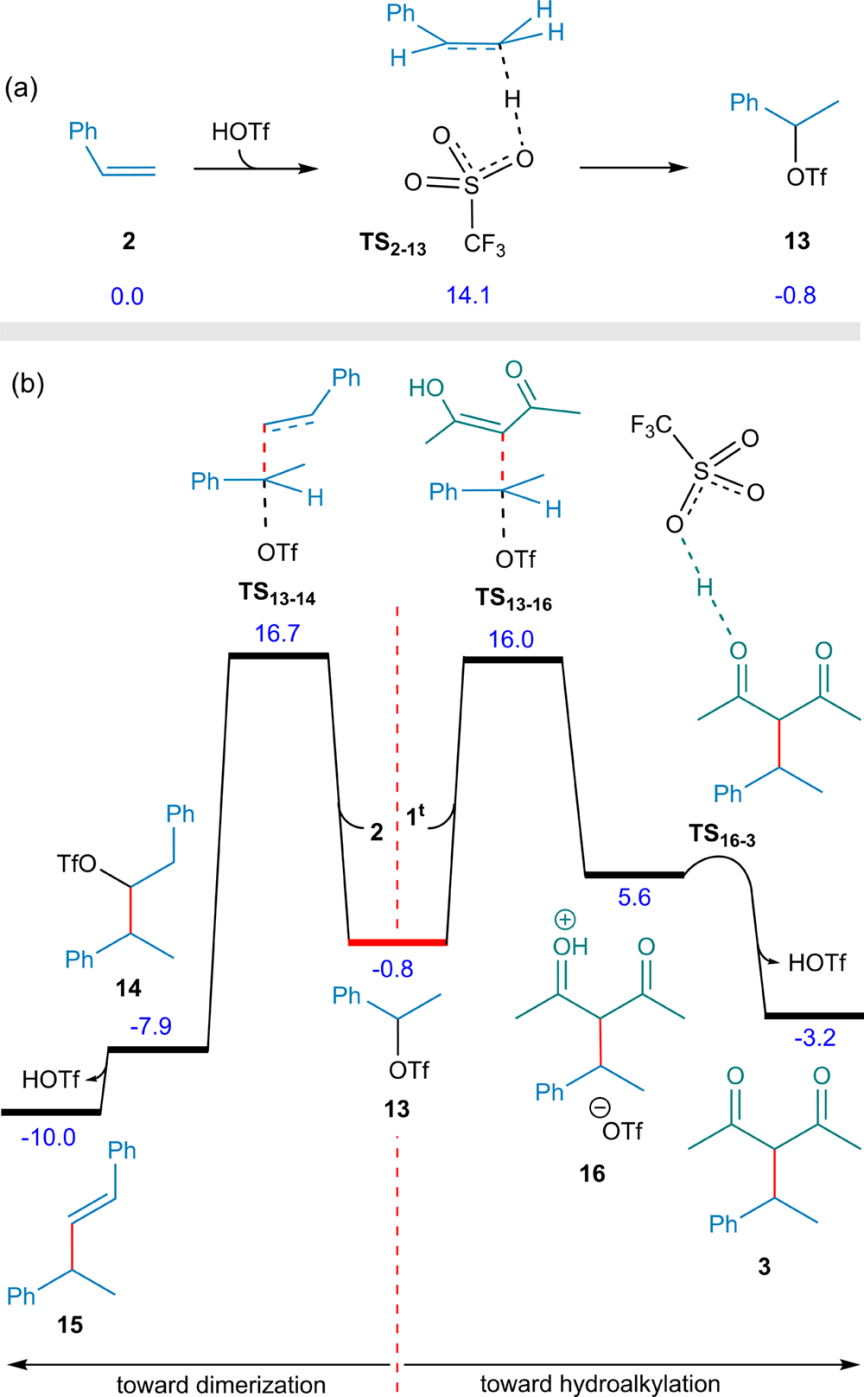
(a) Addition of HOTf to styrene. (b) Right pathway: computed free energy profile leading to the formation of the main product **3**. Left pathway: free energy profile for the competing styrene dimerization pathway. The relative free energies are given in kcal·mol^–1^ where CH_2_Cl_2_ is employed as the solvent.

The left side of Figure 2 depicts a divergent pathway in which another styrene molecule, instead of **1** ^t^, attacks intermediate **13**. The corresponding C–C bond formation transition state (**TS**_**13–14**_) leads to intermediate **14** with a relative free energy of −7.9 kcal mol^–1^; subsequent loss of HOTf further stabilizes this species, yielding **15** at −10.0 kcal mol^–1^. **TS**_**13–14**_ (Δ*G* ^‡^ = 16.7 kcal mol^–1^) lies slightly higher in energy than **TS**_**13–16**_, indicating that both pathways may compete under the reaction conditions. This finding is consistent with previous experimental report of styrene dimerization as a side product under related conditions.^16,17^

Scheme 3 depicts the catalytic cycle proposed for the HOTf-catalyzed hydroalkylation reaction, constructed on the basis of our DFT calculations. On this basis, the hydroalkylation mechanism proceeds through three key steps: (i) electrophilic addition of HOTf to the alkene to generate the benzylic triflate, (ii) nucleophilic attack of the enol form of the diketone accompanied by triflate departure to form the new C–C bond, and (iii) proton transfer that furnishes the hydroalkylation product and regenerates HOTf.

**Scheme 3:**
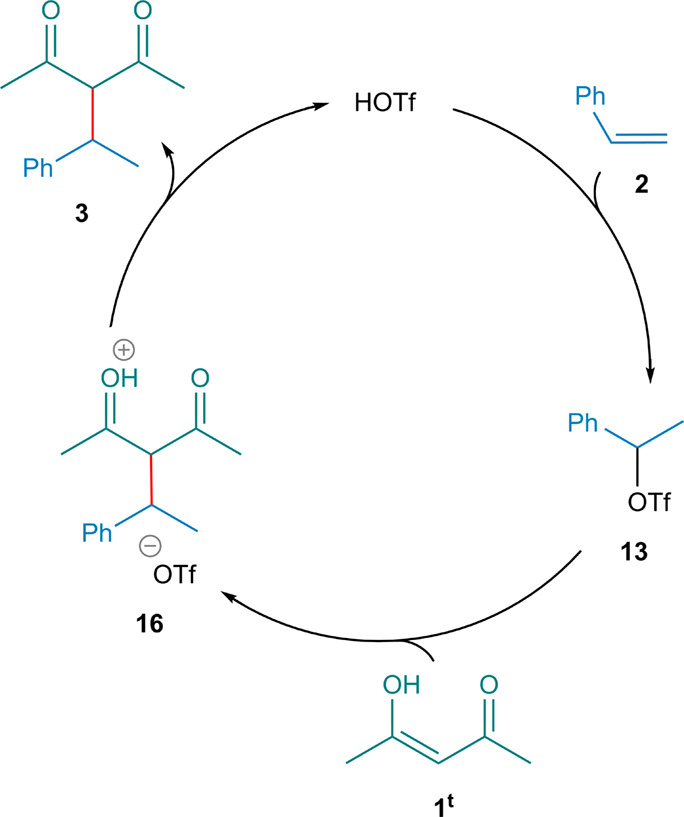
DFT-Derived Catalytic Cycle Proposed for the HOTf-Catalyzed Hydroalkylation of Styrene with 1,3-Diketones

To support the computational findings, the reaction of 2,4-pentanedione **1** with styrene **2** was examined in the presence of catalytic amounts of triflic acid (HOTf) in place of the AuCl_3_/AgOTf catalytic system. Following previously published conditions,^11^ the reaction was first carried out at room temperature with slow addition of styrene over 5 h. The use of catalytic HOTf resulted in an almost 1:1 ratio of the desired product **3** to the styrene dimerization side product **15**, with only a 23% yield of **3** (Table 1, entry 1). Notably, using a higher amount of styrene (2 equiv) led to a slightly improved 32% yield of **3**, while the temperature increase (from r.t. to 45 °C) resulted in a more notable, though still moderate 45% yield of **3** (Table 1, entry 1). In order to further enhance the product formation, the solvent was changed from dichloromethane to toluene and the reaction was heated to 80 °C.^16^ Under these conditions, the use of HOTf resulted in more favorable formation of product **3** (2:1 ratio), with significantly improved 77% yield of **3** (Table 1, entry 2). Notably, performing the reaction in toluene at room temperature led to only 10% yield of **3** (Table 1, entry 2), indicating the crucial role of elevated temperature for the reaction efficiency.

**Table 1:**
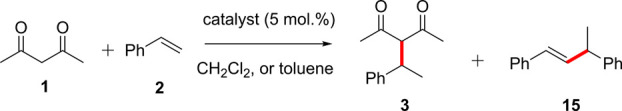
Experimental Evaluation of HOTf, HSO_3_Cl, and MsOH as Catalysts for the Hydroalkylation of Acetylacetone (**1**) with Styrene (**2**)­^a^,^b^

entry	catalyst	solvent and temperature	**3:15** molar ratio	yield of **3** by NMR
1	HOTf	CH_2_Cl_2_, r.t.	1.1:1	23%^c^ ^d^
			0.8:1	32%^d^
		CH_2_Cl_2_, 45 °C	0.9:1	45%
2	HOTf	toluene, 80 °C	2:1	77% (74%)^e^
		toluene, r.t.	2.6:1	10%^d^
3	AuCl_3_/AgOTf^f^	toluene, 80 °C	2:1	81%
4	HSO_3_Cl	toluene, 80 °C	1.4:1	24%^d^
5	MsOH	toluene, 80 °C	1.2:1	14%^d^

a Reaction conditions: **1** (2 mmol, 1 equiv), **2** (4 mmol, 2 equiv), catalyst (0.1 mmol, 5 mol %), CH_2_Cl_2_ (10–12 mL), r.t./45 °C or toluene, 80 °C/r.t., overnight. NMR yields and molar ratios were calculated using triphenylmethane as an internal standard (see the SI for details).

b Reported NMR yields and **3**:**15** product ratios under standard conditions.

c Reaction was performed with 1.5 equiv (3 mmol) of styrene **2**.

d Mostly unreacted starting materials remained, with traces of unidentified side products.

e Isolated yield of product **3**.

f AuCl_3_ (1.7 mol %) and AgOTf (5 mol %) were used (see the SI for details).

Building on these results, a series of Brønsted acids spanning a range of acid strengths was examined computationally in toluene (Table 2). These calculations reveal that sufficiently strong acids can access the low activation barriers required for productive hydroalkylation, whereas acids of moderate or weak strength exhibit substantially higher barriers that are incompatible with the reaction conditions. It should be noted, however, that the p*K*_a_ values of very strong acids are subject to significant uncertainty and depend strongly on the solvent and the acidity scale employed, and therefore should be regarded as qualitative descriptors rather than precise quantitative measures.

**Table 2:** Calculated Activation Barriers for Hydroalkylation and Dimerization Pathways Mediated by Different Acid Catalysts in Toluene

acid	p*K*_a_ (*H*_0_)	TS (hydroalkylation) (kcal mol^–1^)	TS (dimerization) (kcal mol^–1^)
HOTf	–14.7 ^18^	22.8	23.6
HSO_3_Cl	–6.5 ^19^	21.2	21.5
MsOH	–1.9 ^20^	33.3	34.8
HClO_4_	–15.2 ^18^	23.7	24.5
HSO_3_F	(<−14.5)^21^	23.2	23.7
(CF_3_)_3_CSO_3_H	–18.6 ^22^	19.7	20.5
CH_3_C_6_H_4_SO_3_H	–2.8 ^20^	35.7	35.7
HNO_3_	–1.4 ^23^	38.9	40.7
CF_3_COOH	0.2 ^24^	41.1	41.5

Guided by these computational insights, chlorosulfonic acid (HSO_3_Cl) and methanesulfonic acid (MsOH) were selected for experimental evaluation alongside HOTf. However, the use of HSO_3_Cl as a catalyst led to only 24% yield of **3**, although product formation was still favored over dimerization (Table 1, entry 4). Similarly, MsOH showed lower catalytic efficiency, affording only 14% of the product **3**, with nearly equal amounts of **3** and **15** formed (Table 1, entry 5). In addition, AuCl_3_/AgOTf catalytic system^11^ was also tested using toluene at 80 °C conditions, which resulted in a yield of product **3** comparable to that obtained with HOTf (81 vs 77%, Table 1 entries 2 and 3), thus confirming HOTf as an active catalyst for the hydroalkylation reaction.

To provide additional experimental evidence for Au­(I) species and HOTf generation, hydroalkylation of styrene was conducted using AuCl_3_/AgOTf catalysts, followed by treatment with excess of PPh_3_, according to previously reported protocol.^13^ Analysis of the resulting mixture by ^31^P NMR spectroscopy showed the presence of [Au­(PPh_3_)_2_]^+^ (δ 46.43 ppm) and [HPPh_3_]^+^OTf^–^ (δ 3.12 ppm) species (see the SI for the details).

## Conclusion

Our combined computational and experimental study shows that the AuCl_3_/AgOTf catalyzed hydroalkylation of styrenes with 1,3-diketones does not proceed through the Au­(OTf)_3_ initiated benzylic carbocation mechanism previously proposed for this transformation. Instead, our calculations demonstrate that Au­(OTf)_3_ is thermodynamically predisposed to undergo processes that lead to the formation of HOTf, and that the resulting Au­(III) intermediate can further evolve to Au­(I). The pathway leading to HOTf formation from the relevant LAu­(OTf)_3_ intermediates is both kinetically and thermodynamically more favorable than the route that generates the benzylic cation, indicating that the latter is unlikely to act as the true on-cycle catalyst. Based on these findings, we propose an alternative Brønsted acid mechanism in which HOTf generated in situ functions as the active catalyst. In this cycle, HOTf activates styrene through electrophilic addition to form the corresponding benzylic triflate. This intermediate is then intercepted by the enol tautomer of the 1,3-diketone in an S_N_2 type C–C bond forming step, after which proton transfer from the protonated enol to the triflate anion regenerates HOTf. This mechanistic picture also accounts for the competing dimerization of styrene, which arises from reaction of the same benzylic intermediate with another styrene molecule and is observed experimentally as a significant side process.

Extending our analysis to other Brønsted acids further supports this conclusion. Both the computed activation barriers and our experimental tests show that, in addition to HOTf, other strong Brønsted acids are also capable of catalyzing the hydroalkylation reaction, whereas weaker acids are ineffective. These trends indicate that, in the present system, the overall reactivity is governed primarily by Brønsted acidity.

Together, these findings refine the mechanistic understanding of the AuCl_3_/AgOTf system studied here and support the conclusion that the reaction operates through in situ generation of a strong Brønsted acid, with concomitant reduction of Au­(III) to Au­(I), rather than through a discrete Au-centered or free carbocation pathway under the examined conditions.

## Computational Details

Gaussian 16 ^25^ was used to fully optimize all the structures reported in this paper at the M06 level of theory.^26^ For all the calculations, solvent effects were considered using the SMD solvation model.^27^ The SDD basis set^28,29^ with effective core potential (ECP) was chosen to describe gold, and silver. The 6–31G­(d) basis set was used for other atoms.^30^ This basis set combination will be referred to as BS1. Frequency calculations were carried out at the same level of theory as those for the structural optimization. Transition structures were located using the Berny algorithm. IRC calculations were used to confirm the connectivity between transition structures and minima.^31,32^ To further refine the energies obtained from the SMD/M06/SDD,6–31G­(d) calculations, we carried out single-point energy calculations using the M06 functional method with the SMD solvation model in dichloromethane along with a larger basis set (BS2) for all the optimized structures. BS2 utilizes the def2-TZVP basis set^33^ on all atoms. The tight convergence criterion and ultrafine integral grid were exploited to increase the accuracy of the calculations. The free energy for each species in solution was calculated using the following formula:

$$G=E(\mathrm{BS}2)+G(\mathrm{BS}1)-E(\mathrm{BS}1)+\Delta G^{1\mathrm{atm}\to 1M}$$ 1

where Δ*G* ^1atm→1M^ = 1.89 kcal/mol is the free-energy change for compression of 1 mol of an ideal gas from 1 atm to the 1 M solution phase standard state.

## Supplementary Material



## Notes

Additional computational and experimental details, Cartesian coordinates of optimized structures, and relative energy values (PDF)
