# Supplementary material for: Identification of HOTf-Driven Brønsted Acid Catalysis in the AuCl3/AgOTf System for the Hydroalkylation of Styrene
Source: Inorg Chem. 2026 May 5;65(19):10885–91. doi: 10.1021/acs.inorgchem.6c01693 (PMC13188052; doi:10.1021/acs.inorgchem.6c01693)
Supplement: Supplementary file 1 [file ic6c01693_si_001.pdf]

Supporting Information for:

Identification of HOTf-Driven Brønsted Acid Catalysis in the $AuCl_3$/AgOTf System for Hydroalkylation of Styrene

Amir Mahdian[1], Tatsiana Nikonovich[1], Sandra Kaabel[1*], Kari Laasonen[1*], and Kaveh Farshadfar[1*]

[1]Department of chemistry and material science, School of chemical engineering, Aalto University, 02150 Espoo, Finland

Corresponding Authors

Kaveh Farshadfar — Email: Kaveh.Farshadfar@Aalto.fi

Kari Laasonen — Email: Kari.Laasonen@Aalto.fi

Sandra Kaabel — Email: Sandra.Kaabel@aalto.fi

Content:

General information and full experimental details (page S2)

Effect of solution components on catalyst speciation (page S6)

Energies and Cartesian coordinates of the calculated structures (page S10)

General information

All chemicals and solvents were used as obtained from commercial suppliers (Sigma-Aldrich, TCI Chemicals, Fisher Scientific, BLD Pharm). Dry dichloromethane and toluene were obtained by passing deoxygenated solvent through activated alumina columns (MBraun SPS-800 series Solvent Purification System).

Thin-layer chromatography (TLC) was carried out using Merck silica gel 60 plates (F254) and visualized with UV light (254 nm) and phosphomolybdic acid (PMA) stain. Merck silica gel 60 (Merck, 40–63 μm) was used for column chromatography. ^{1}H NMR (400 MHz), ^{13}C NMR (100.6 MHz) and ^{31}P NMR (162 MHz) spectra were recorded on a Bruker Avance NEO 400 MHz spectrometer. All chemical shifts are reported in ppm units and are referenced to the residual solvent signal ($CDCl_3$: δ ^{1}H 7.26 and δ ^{13}C 77.16 ppm) or tetramethylsilane (δ ^{1}H 0.00 and δ ^{13}C 0.00 ppm) for ^{1}H and ^{13}C respectively. ^{31}P NMR spectra were referenced with an internal TMS ^{1}H resonance (absolute referencing). Triphenylmethane was used as an internal standard to determine yields by quantitative ^{1}H NMR spectroscopy.

Reaction conditions using dichloromethane (adapted from Yao et al.[1]): HOTf (0.1 mmol, 5 mol %, 9 μL) was introduced (using 10 μL Shimadzu microsyringe) into the solution of 2,4-pentanedione **1** (206 μL, 2 mmol, 1 equiv.) in 4 mL of dry dichloromethane under an inert atmosphere of Ar. To the obtained mixture the solution of styrene **2** (344 μL, 3 mmol, 1.5 equiv. *or* 460 μL, 4 mmol, 2 equiv.) in 6 mL of dry dichloromethane (8 mL for 2 equiv. of styrene **2**) was added dropwise in 5 hours. The mixture was stirred at room temperature or at 45 ºC for overnight, then concentrated under reduced pressure and analyzed by ^{1}H NMR spectroscopy using triphenylmethane as an internal standard (Table S1, entries 1 and 2).

Reaction conditions using toluene (adapted from Yao et al.[2]: A catalyst (0.1 mmol, 5 mol %) was introduced (using 10 μL Shimadzu microsyringe) into the solution of 2,4-pentanedione **1** (206 μL, 2 mmol, 1 equiv.) and styrene **2** (460 μL, 4 mmol, 2 equiv.) in 4 mL of dry toluene under an inert atmosphere of Ar. The mixture was stirred at room temperature or at 80°C for overnight, then concentrated under reduced pressure and analyzed by ^{1}H NMR spectroscopy using triphenylmethane as an internal standard (Table S1, entries 3–6).

Reaction conditions using toluene and $AuCl_3$/TfOH as catalysts (adapted from Yao et al. [1]): $AuCl_3$ (0.034 mmol, 1.7 mol %, 10.2 mg) and AgOTf (0.1 mmol, 5 mol %, 26 mg) were added into 4 mL of dry toluene under an inert atmosphere of Ar. The mixture was stirred at room temperature for 2 hours. Then 2,4-pentanedione **1** (206 μL, 2 mmol, 1 equiv.) and styrene **2** (460 μL, 4 mmol, 2 equiv.) were added into the solution of catalysts. The mixture was stirred at 80 ºC for overnight, then concentrated under reduced pressure and analyzed by ^{1}H NMR spectroscopy using triphenylmethane as an internal standard (Table S1, entry 7).

Table S1.

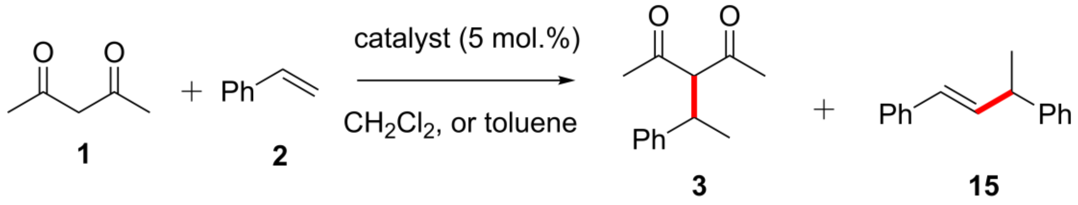

	Catalyst	Solvent and temperature	Yield of **3** by NMR[a]	Yield of **15** by NMR[a]	**3**:**15** ratio (mmol)
1	TfOH, 9 μL	CH_2Cl_2, r.t.	23%[b,c]	41%	1.1 : 1
			32%[c]	76%[b]	0.8 : 1[b]
2	TfOH, 9 μL	CH_2Cl_2, 45 °C	45%	96%	0.9 : 1
3	TfOH, 9 μL	Toluene, 80 °C	77% (74%)[d]	78%	2 : 1
4	TfOH, 9 μL	Toluene, r.t.	10%[c]	8%	2.6 : 1
5	HSO_3Cl, 7 μL	Toluene, 80 °C	24%[c]	35%	1.4 : 1
6	MsOH, 6.5 μL	Toluene, 80 °C	14%[c]	23%	1.2 : 1
7	$AuCl_3$/AgOTf	Toluene, 80 °C	81%	83%	2 : 1

[a] Yields were determined by ^{1}H NMR in $CDCl_3$ using triphenylmethane as an internal standard (characteristic signals of product **3** at 4.04 (d, *J* = 11.3 Hz, 1H) and side product **15** at δ 6.42 – 6.37 (m, 2H) were integrated), based on **2** (2 mmol) for **3** (2 mmol, teor.) and **15** (1 mmol, teor.). [b] Reaction was performed with 1.5 equiv. of styrene **2**. [c] Mostly unreacted starting materials remained, with traces of unidentified by-products. [d] Isolated yield of product **3** is indicated in the brackets.

The product **3** (Table S1, entry 3) was purified by silica gel column chromatography (4 to 5% EtOAc/hexane) and obtained as a pale-yellow oil (362 mg, 74% yield (considering 84% purity by ^{1}H NMR using triphenylmethane as an internal standard)).

3-(1-phenylethyl)pentane-2,4-dione 3. ^{1}H NMR (400 MHz, $CDCl_3$): δ 7.32-7.25 (m, 2H), 7.23-7.16 (m, 3H), 4.04 (d, *J* = 11.3 Hz, 1H), 3.59 (dq, *J* = 11.3, 6.9 Hz, 1H), 2.27 (s, 3H), 1.83 (s, 3H), 1.21 (d, *J* = 6.9 Hz, 3H). ^{13}C NMR (101 MHz, $CDCl_3$): δ 203.62, 203.57, 143.14, 128.93, 127.42, 127.38, 127.10, 76.83, 40.55, 29.91, 29.81, 20.97. Spectral data are in agreement with previously reported.[1]

Characteristic peaks of **but-1-ene-1,3-diyldibenzene 15.** ^{1}H NMR (400 MHz, $CDCl_3$): δ 6.42 – 6.37 (m, 2H), 3.68 – 3.59 (m, 1H), 1.46 (d, *J* = 7.1 Hz, 3H).[3]

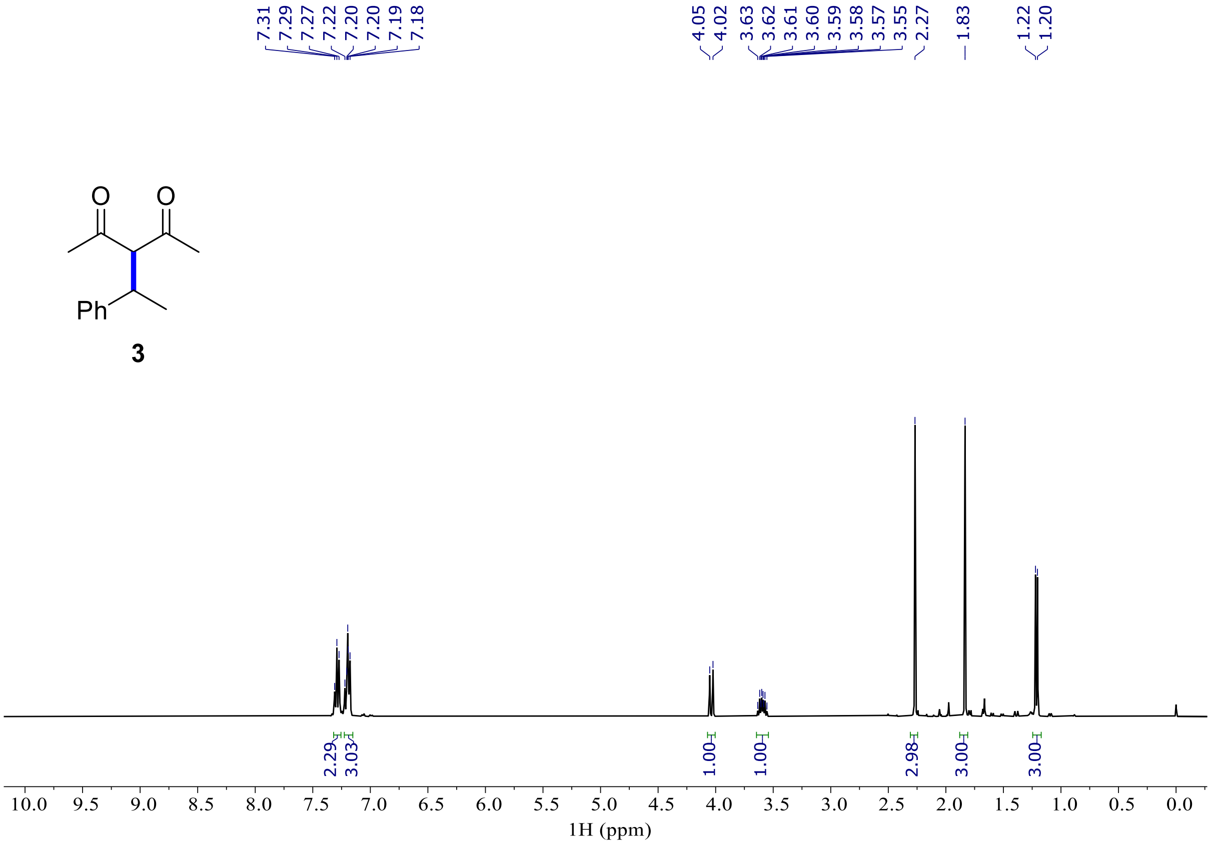
7.31
7.29
7.27
7.22
7.20
7.20
7.19
7.18
4.05
4.02
3.63
3.62
3.61
3.60
3.59
3.58
3.57
3.55
2.27
1.83
1.22
1.20
O
O
Ph
3
2.29
3.03
1.00
1.00
2.98
3.00
3.00
1H (ppm)

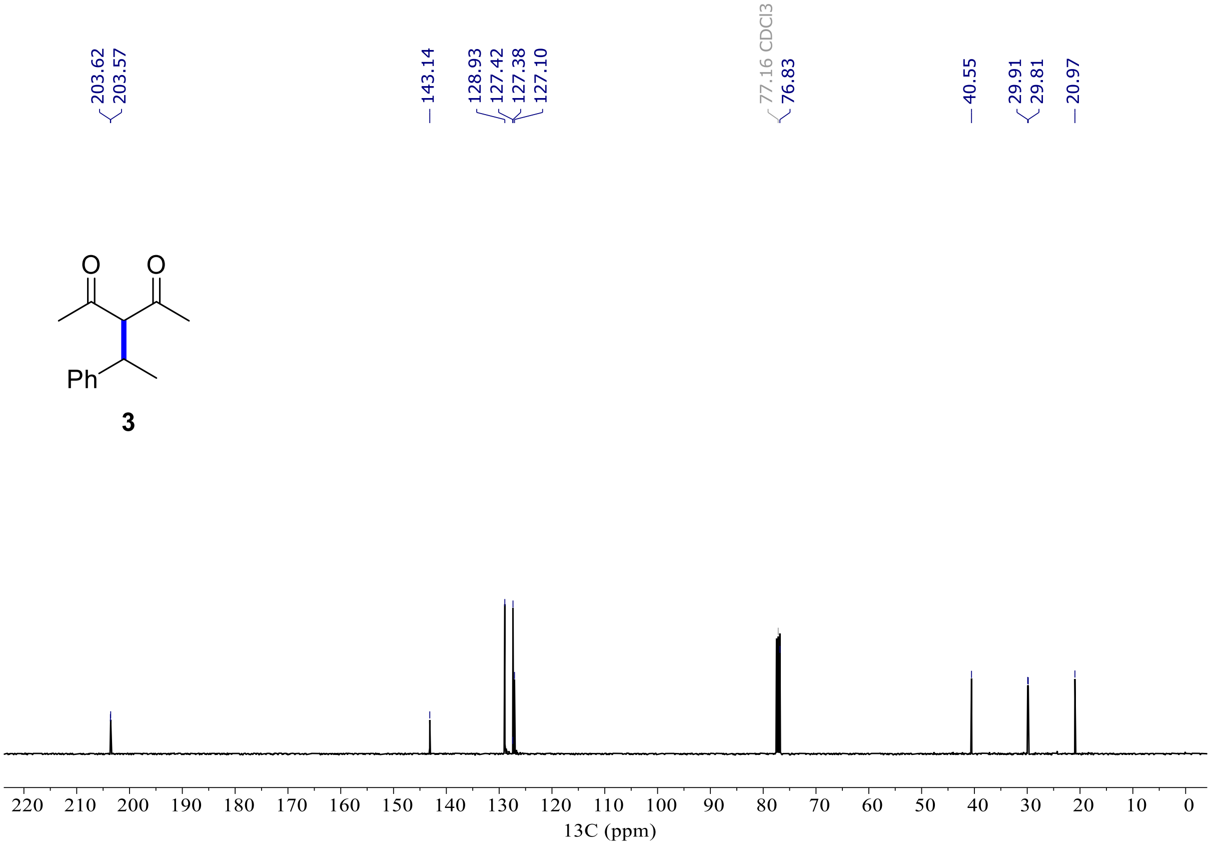
203.62
203.57
143.14
128.93
127.42
127.38
127.10
77.16 CDCl3
76.83
40.55
29.91
29.81
20.97
O
O
Ph
3
13C (ppm)

$[Au(PPh_3)_2]^+$ Complex formation – in situ formation of Au(I) cation

To identify the presence of Au(I) cations under catalytic $AuCl_3$/AgOTf reaction conditions, the previously published protocol [4] was followed. The catalysts $AuCl_3$ (76 mg, 0.25 mmol, 50 mol%) and AgOTf (193 mg, 0.75 mmol, 150 mol%) were stirred in CH_2Cl_2 (3.0 mL) for 2 hours at room temperature under an inert atmosphere of Ar. Then 2,4-pentanedione **1** (51 µL, 0.5 mmol, 1 equiv.) was added, followed by styrene **2** (63 µL, 0.55 mmol, 1.1 equiv.) in 0.5 mL of CH_2Cl_2. The reaction mixture was heated at 45 °C for 24 hours under an inert atmosphere of Ar. Then, PPh_3 (262 mg, 1 mmol, 4 equiv. wrt Au, dissolved in 1 mL of CH_2Cl_2) was added to the cooled reaction mixture and stirred at room temperature for 2 hours, then concentrated under reduced pressure and analyzed by ^{31}P NMR spectroscopy (Figure S1).

$^{31}P\{^1H\}$ NMR (162 MHz, CD_2Cl_2) δ 46.43 ppm ($[Au(PPh_3)_2]^+$), 34.51 ppm ($[Au(PPh_3)]^+$). The spectroscopic data are in agreement with previously reported. [4]

$[HPPh_3]^+OTf^-$ Standard synthesis

HOTf (0.1 mmol, 9 µL) was introduced (using 10 µL Shimadzu microsyringe) into the solution of PPh_3 (26 mg, 0.1 mmol) in 2 mL of dry dichloromethane under an inert atmosphere of Ar. The reacrion mixture was stirred at room temperature for 30 minutes, then concentrated under reduced pressure and analyzed by ^{31}P NMR spectroscopy (Figure S1).

$^{31}P\{^1H\}$ NMR (162 MHz, CD_2Cl_2) δ 3.12 ppm (s).

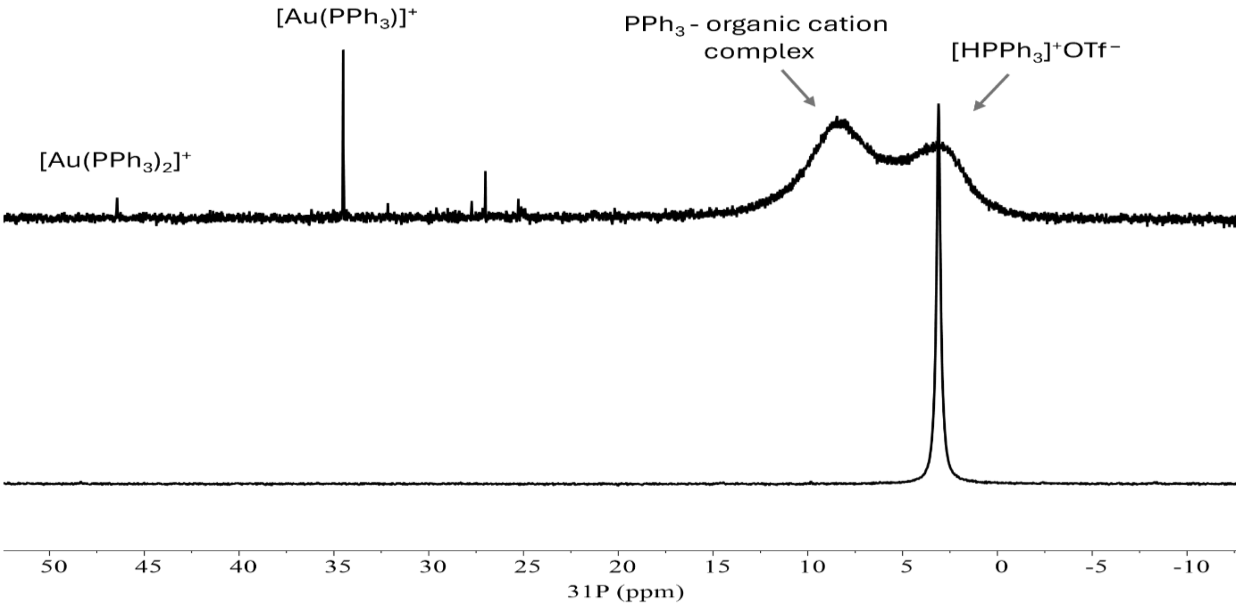

Figure S1. $^{31}P\{^1H\}$ NMR spectra of the crude reaction isolate (top spectra) containing $[Au(PPh_3)_2]^+$, $[Au(PPh_3)]^+$, and $[HPPh_3]^+OTf^-$ species *vs* a standard sample of $[HPPh_3]^+OTf^-$ (bottom spectra).

Effect of solution components on catalyst speciation

Although rigorously dried solvents were used in the experimental section, we consider here the possible influence of trace amounts of water present as adventitious moisture in the reaction medium. Specifically, we evaluate its effect on both the $AuCl_3$/AgOTf-catalyzed reaction and the HOTf-mediated pathway. Furthermore, the potential interactions of silver chloride with key intermediates in the reaction are also examined.

The computational results indicate that water exhibits significantly weaker coordination to the Au(III) center than styrene (reaction 1, Figure S2). Moreover, water does not form a strong interaction with the Au(III) species prior to its reduction to Au(I) (reaction 1, Figure S2). Reaction 3 shows that water can form a slightly stable adduct with HOTf, with a relative free energy of −1.4 kcal mol^{-1}. In addition, reaction 4 demonstrates that water does not assist the key C–C bond-forming step of this transformation, as its involvement in proton shuttling increases the transition-state energy by 5.7 kcal mol^{-1}.

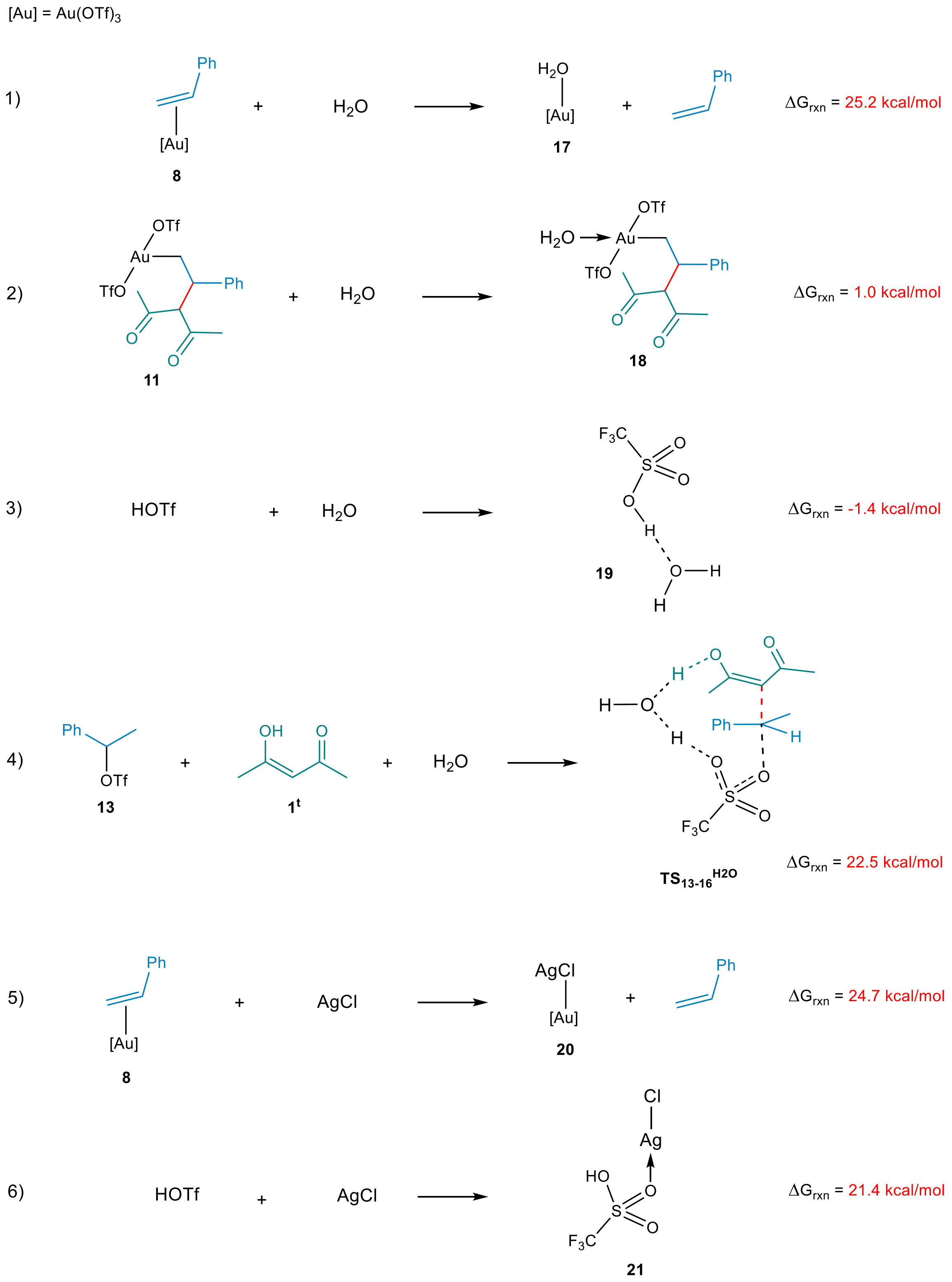

Figure S2. Relative free energies of key structures upon interaction with water or AgCl. All calculations were carried out in CH_2Cl_2 as the solvent.

Since AgCl precipitates under the reaction conditions and is not present as a free species in solution, its potential involvement in the reaction requires consideration of the energetic cost associated with extracting a unit from the solid phase. To estimate this quantity, a series of finite AgCl clusters of the form $(AgCl)_n$ (n = 1, 4, 6, 12, and 18) were constructed and fully optimized using the computational method employed in this study. The Gibbs free energy of each cluster was calculated and normalized per formula unit to obtain G(n)/n.

To estimate the limiting value of G(n)/n as n increases, the data were fitted using a first-order asymptotic model of the form:

$G(n)/n = G_\infty + a/n$

where G_∞ represents the limiting value as $n \rightarrow \infty$, and a is a fitting parameter. This expression provides a first-order approximation for the deviation from the limiting value and enables extrapolation to infinite n. A linear fit of G(n)/n as a function of 1/n was performed, and the extrapolated value at $1/n \rightarrow 0$ provides an estimate of G_∞. This limiting value was then used as a reference to estimate the energetic cost associated with making an AgCl unit available for interaction with key intermediates in the reaction.

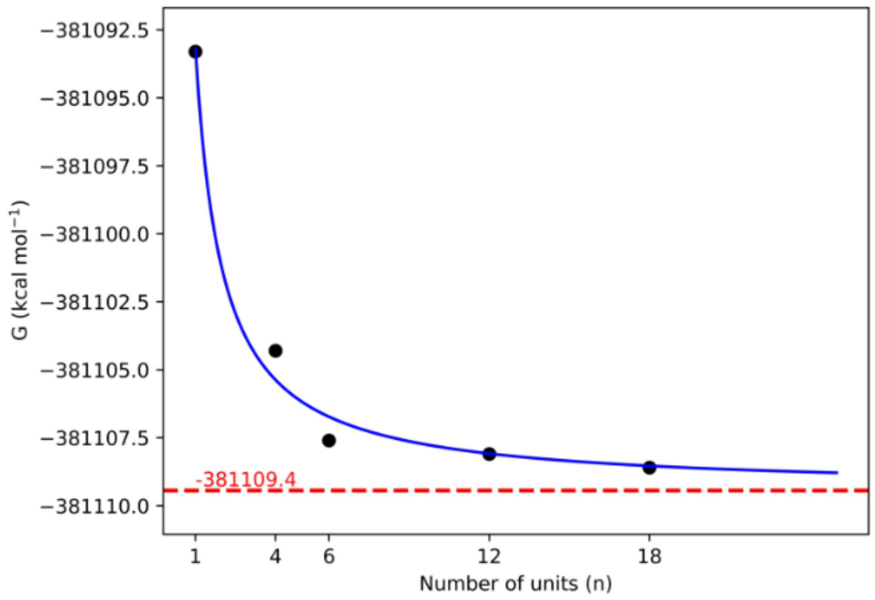

Figure S3. Extrapolation of G(n)/n as a function of cluster size (n) using a first-order asymptotic model ($G(n)/n = G_\infty + a/n$). The dashed line indicates the extrapolated limiting value (G_∞) at $n \rightarrow \infty$, used to estimate the Gibbs free energy per AgCl unit in the solid phase.

To evaluate whether heterobimetallic Au–Ag interactions can be established, styrene in the complex 8 was replaced with AgCl. Geometry optimization was initiated from a structure in which the silver atom was positioned in proximity to the gold center, with a linear Au–Ag–Cl arrangement. During the optimization process, however, the AgCl unit reoriented, coordinating to the gold center through the chlorine atom rather than maintaining direct Au–Ag interaction. This substitution of styrene by AgCl was found to be highly unfavorable, with a relative free

energy increase of 24.7 kcal mol^{-1}. (reaction 5, Figure S1). Reaction 6 indicates that AgCl does not establish a significant interaction with HOTf.

Overall, the computational results indicate that precipitated silver salts present in solution when using the $AuCl_3$/AgOTf catalytic system do not exert a significant direct influence on the reaction. In addition, the presence of trace amounts of water appears to have a negligible effect on the stability of key species along the reaction pathway.

Table S2. Total potential (E), and Gibbs free energies (G) of all structures optimized at the SMD/M06-2X/6-31G(d) level of theory along with the total potential energies calculated by SMD/M06-2X/def2-TZVP//SMD/M06-2X/6-31G(d) and Cartesian coordinates for all of the calculated structures.

1
E (SMD/M06-2X/6-31G(d)) = -345.579038983
G (SMD/M06-2X/6-31G(d)) = -345.491616
E (SMD/M06-2X/def2-TZVP//SMD/M06-2X/6-31G(d)) = -345.722439640

C	4.94202000	-3.13067100	-0.26603400
H	4.16715800	-3.87761200	-0.49040700
H	5.46223400	-3.40941100	0.65570700
H	5.64545900	-3.13531100	-1.10920100
C	4.31299600	-1.77789300	-0.13152000
O	4.31741000	-1.14726000	0.90727200
C	3.66131400	-1.21649500	-1.37663500
C	4.67558400	-0.58038900	-2.30905800
C	4.09761600	0.19121200	-3.45656300
H	3.33203300	-0.39746200	-3.97886400
H	3.59779800	1.09173500	-3.07255200
H	4.88682000	0.48782700	-4.15440100
O	5.87293700	-0.68581000	-2.12367400
H	3.13760400	-2.00237600	-1.94383500
H	2.91362700	-0.46124700	-1.09717500

1^{t}
E (SMD/M06-2X/6-31G(d)) = -345.580237210
G (SMD/M06-2X/6-31G(d)) = -345.489334
E (SMD/M06-2X/def2-TZVP//SMD/M06-2X/6-31G(d)) = -345.724995644

C	4.42221500	-3.26366600	0.04309900
H	3.35094500	-3.20764700	0.27999100
H	4.98267100	-3.52034300	0.94738900
H	4.54067900	-4.06269400	-0.70137800
C	4.89852400	-1.95657800	-0.51608400
O	5.80698400	-1.32368900	0.05749600
C	4.27140700	-1.47590400	-1.71962000
C	4.65128600	-0.29097500	-2.28895400
C	4.04863100	0.26317100	-3.52610700
H	4.82570500	0.40895100	-4.28835700
H	3.27246300	-0.39584300	-3.92637500
H	3.61287600	1.25019800	-3.32101900
O	5.60419000	0.47186000	-1.77357500
H	5.92480400	-0.00666700	-0.95279100
H	3.48309300	-2.06080400	-2.18763300

2

E (SMD/M06-2X/6-31G(d)) = -309.393643865

G (SMD/M06-2X/6-31G(d)) = -309.292119

E (SMD/M06-2X/def2-TZVP//SMD/M06-2X/6-31G(d)) = -309.513483809

C	2.88710000	-2.15961900	-4.73772500
C	2.74638200	-0.91917700	-5.21318400
C	2.92641100	-3.40625000	-5.51008200
C	3.09562100	-4.61692400	-4.82585400
C	2.80217700	-3.44617400	-6.90689700
C	3.14068800	-5.82852900	-5.50739400
H	3.19329800	-4.59982400	-3.73953900
C	2.84684000	-4.65464600	-7.58869600
H	2.66804600	-2.52104000	-7.46699200
C	3.01648500	-5.85193100	-6.89313200
H	3.27341500	-6.75693800	-4.95361100
H	2.74827200	-4.66470100	-8.67344800
H	3.05081700	-6.79756600	-7.43197400
H	2.98711700	-2.29332400	-3.65696700
H	2.64231400	-0.69916600	-6.27586800
H	2.73081000	-0.06258900	-4.54183700

3

E (SMD/M06-2X/6-31G(d)) = -655.010087673

G (SMD/M06-2X/6-31G(d)) = -654.794063

E (SMD/M06-2X/def2-TZVP//SMD/M06-2X/6-31G(d)) = -655.265150941

C	-11.91757900	3.96137500	-7.83843100
C	-11.99325000	3.82058800	-9.22304500
C	-11.17562900	2.89411400	-9.86229200
C	-10.28250200	2.11777100	-9.12617100
C	-10.19028800	2.25557200	-7.73897700
C	-11.02548600	3.18412600	-7.10801300
H	-12.55973900	4.67638600	-7.32557900
H	-12.69092400	4.42690100	-9.79885400
H	-11.23033000	2.77185300	-10.94334500
H	-9.64630500	1.40221500	-9.64657200
H	-10.96854400	3.29647600	-6.02333200
C	-9.17868200	1.48937200	-6.91154400
H	-9.63327500	1.29296600	-5.92805400
C	-8.78104400	0.14763000	-7.51224000
H	-8.15915600	-0.41557600	-6.80782400
H	-9.67644400	-0.45058800	-7.72681500
H	-8.21219200	0.25414900	-8.44316600
O	-7.38093200	0.90592300	-4.82665600
C	-7.02522800	1.80909800	-5.55779600
C	-5.67046600	2.44268700	-5.43080100
H	-5.71762500	3.53143300	-5.56988000
H	-5.23443100	2.20434800	-4.45564200

H	-5.01372800	2.04445200	-6.21623000
C	-7.96516800	2.40488800	-6.60126300
C	-7.23744100	2.81982100	-7.87261700
O	-6.51163300	2.03245600	-8.45361200
C	-7.45808600	4.22021600	-8.35703900
H	-8.53092400	4.45372500	-8.41125000
H	-7.02311000	4.92330000	-7.63035500
H	-6.98819000	4.37096400	-9.33398600
H	-8.35641600	3.32032800	-6.12030000

4

E (SMD/M06-2X/6-31G(d)) = -3364.93794267

G (SMD/M06-2X/6-31G(d)) = -3364.789622

E (SMD/M06-2X/def2-TZVP//SMD/M06-2X/6-31G(d)) = -3366.03449474

C	-1.79838400	3.47213400	1.01150400
H	-2.74161400	3.89762000	0.63024400
H	-1.32747300	4.17421800	1.70373900
H	-2.07468200	2.53524900	1.51089800
C	-0.94082400	3.20794900	-0.14112800
O	0.08832100	3.93929200	-0.30136200
C	-1.25185100	2.14817100	-1.11505400
C	-0.62065400	2.28874100	-2.48096200
O	0.38284100	2.98612400	-2.61624700
C	-1.26022400	1.56291400	-3.60440600
H	-2.21190900	2.06020800	-3.84474300
H	-0.61212200	1.57364500	-4.48476800
H	-1.50938900	0.53047400	-3.32267200
H	-2.32188600	1.91565200	-1.13603900
H	0.53216600	3.70324100	-1.19411800
Au	-0.41136500	0.33015400	-0.38883900
O	0.40520300	-1.52659700	0.20130600
S	0.69658000	-2.55526900	-0.90841200
O	0.56160900	-3.90556700	-0.39613900
O	0.08106100	-2.18362800	-2.17539000
C	2.49996400	-2.29099200	-1.15101800
F	3.14739100	-2.51568800	-0.02180000
F	2.93176500	-3.12196900	-2.08428400
F	2.71473800	-1.04216300	-1.54163900
O	2.77133900	2.88237800	0.97353300
S	1.90225500	1.74209500	1.17869500
O	0.80079100	1.79374600	2.12813800
O	1.40744700	1.24880500	-0.22111300
C	3.00973700	0.37107500	1.72510600
F	3.63651400	0.78247200	2.81256700
F	2.30141500	-0.70382000	2.00015100
F	3.88404500	0.10980000	0.77389800
O	-3.48866800	0.77083000	0.92726500

S	-3.33886000	-0.55920400	0.35789700
O	-4.47812400	-1.26602000	-0.18710600
O	-2.19374100	-0.62041300	-0.69654000
C	-2.67102900	-1.60610400	1.72102000
F	-3.60747400	-1.72312500	2.64261500
F	-2.34425000	-2.79311700	1.25506600
F	-1.60488800	-1.01574100	2.24502100

$TS_{4\text{-}5}$

E (SMD/M06-2X/6-31G(d)) = -3674.32433435

G (SMD/M06-2X/6-31G(d)) = -3674.056789

E (SMD/M06-2X/def2-TZVP//SMD/M06-2X/6-31G(d)) = -3675.53254487

C	-1.79597500	3.22544100	1.62828400
H	-2.86668500	3.47168700	1.57106100
H	-1.31051800	3.87736800	2.35999600
H	-1.72950900	2.17779500	1.94760400
C	-1.18380300	3.42452600	0.28109600
O	-0.44547100	4.34827100	0.00540300
C	-1.61167300	2.42031700	-0.77194000
C	-1.48279900	2.77892200	-2.21257100
O	-0.54020300	3.42477700	-2.70602600
C	-2.55828000	2.30547300	-3.11455600
H	-3.50754400	2.77269100	-2.81341500
H	-2.33396100	2.54642700	-4.15642600
H	-2.69774300	1.22072000	-2.99531700
H	-2.62101700	2.05124000	-0.55943500
H	0.48765200	3.91880100	-2.19241300
Au	-0.49780100	0.63446100	-0.65248200
O	0.56757900	-1.22820300	-0.63167200
S	0.77032500	-1.89782600	-1.99314800
O	0.80046600	-3.34447200	-1.87039700
O	-0.03234500	-1.27609100	-3.04198800
C	2.49768600	-1.39226200	-2.36098400
F	3.30607400	-1.79313200	-1.39427700
F	2.87893800	-1.93293700	-3.50735000
F	2.55491400	-0.07083900	-2.46200200
O	2.65395700	3.18295300	0.84822600
S	1.88513700	1.95198300	0.89557800
O	0.94851000	1.70066200	1.97842700
O	1.23966800	1.71321000	-0.50156000
C	3.15180800	0.61420100	0.99335400
F	3.88458800	0.84096200	2.07021300
F	2.57088000	-0.56339400	1.09373700
F	3.91809400	0.64929400	-0.08131300
O	-3.51744500	0.47886200	0.97288600
S	-3.22873100	-0.71393300	0.19309500
O	-4.30379000	-1.52049800	-0.34602900

O	-2.21246100	-0.44596200	-0.95317600
C	-2.26193300	-1.81259600	1.31429800
F	-3.03748000	-2.15541000	2.32517500
F	-1.86816000	-2.88670800	0.66226700
F	-1.20394100	-1.15446500	1.77541200
C	5.11103100	2.03674200	-2.56593500
C	4.07829500	2.92490600	-2.31165900
C	3.26900200	3.38307300	-3.36778600
C	3.51845100	2.92388500	-4.67323200
C	4.53914300	2.01794400	-4.91891900
C	5.33831200	1.57895100	-3.86494100
H	5.74652900	1.69597400	-1.75113900
H	3.90841700	3.28591200	-1.29772100
H	2.89644400	3.28847900	-5.49054400
H	4.72121500	1.66118900	-5.93052800
H	6.15069700	0.87980400	-4.05631600
C	2.21321200	4.34581000	-3.17113900
H	1.79220500	4.76154300	-4.09236700
C	1.62716300	4.72379500	-1.98972100
H	2.03544800	4.40802500	-1.02880600
H	1.03077500	5.63986400	-1.98475500

5

E (SMD/M06-2X/6-31G(d)) = -3364.51517598
G (SMD/M06-2X/6-31G(d)) = -3364.381579
E (SMD/M06-2X/def2-TZVP//SMD/M06-2X/6-31G(d)) = -3365.61231139

C	-0.48663100	-3.32218900	1.65317500
H	-0.22425900	-4.33684900	1.31810100
H	-1.17214500	-3.38224900	2.50305000
H	0.45147100	-2.83903100	1.95149000
C	-1.13574600	-2.59679400	0.52114900
O	-2.32275000	-2.33659600	0.47529000
C	-0.24346900	-2.23826500	-0.65521100
C	-0.97061600	-1.96820100	-1.95462700
O	-0.84474900	-0.90539500	-2.53418400
C	-1.80543200	-3.09225300	-2.47998900
H	-2.76833100	-3.08832800	-1.95197100
H	-1.98461500	-2.95008100	-3.55060300
H	-1.33446800	-4.06678700	-2.29807600
H	0.54493100	-2.99109000	-0.79910700
Au	0.85053200	-0.49566800	-0.28053900
O	1.99054800	1.39423200	-0.12353800
S	2.40531900	2.02976300	-1.43905500
O	3.62130500	2.81886500	-1.30206800
O	2.27346300	1.12839100	-2.57986000
C	1.06143800	3.25611000	-1.68305800
F	1.09029700	4.17446200	-0.72924900

F	1.21606400	3.85166500	-2.85824200
F	-0.11597000	2.64259700	-1.66179500
O	-2.61852200	1.09472700	1.86531200
S	-1.24401400	0.63769800	1.78358100
O	-0.78085000	-0.46802600	2.60787700
O	-0.86822800	0.47879200	0.29237000
C	-0.22025600	2.07408400	2.32112800
F	-0.68164500	2.48796500	3.49220200
F	1.04208800	1.70742800	2.46203300
F	-0.30822300	3.05613500	1.44272800
O	2.57806700	-3.36305100	0.65611100
S	3.38810000	-2.38193300	-0.05043100
O	4.56209900	-2.80779600	-0.78754500
O	2.55668200	-1.44250200	-0.94924500
C	4.00807400	-1.23630400	1.25328300
F	4.84243200	-1.90431800	2.03096800
F	4.63493900	-0.21706900	0.69916400
F	2.99504400	-0.78822600	1.98854100

6

E (SMD/M06-2X/6-31G(d)) = -309.81453205

G (SMD/M06-2X/6-31G(d)) = -309.700802

E (SMD/M06-2X/def2-TZVP//SMD/M06-2X/6-31G(d)) = -309.927190162

C	-4.89168100	0.41230800	-0.22045600
C	-6.24939300	0.13664200	-0.33269200
C	-6.81497000	-0.50029600	-1.47480700
C	-7.09661200	0.51450100	0.74959200
C	-8.16861700	-0.73815700	-1.52209000
H	-6.17840000	-0.79929600	-2.30391700
C	-8.45039100	0.27099500	0.68907800
H	-6.65158700	0.99774500	1.61808300
C	-8.98043500	-0.35243100	-0.44548500
H	-8.61251200	-1.22272300	-2.38771000
H	-9.10252000	0.55751900	1.50976100
H	-10.05088600	-0.54472400	-0.49493900
H	-4.57576000	0.89975200	0.70681400
C	-3.83076500	0.13379300	-1.17628100
H	-3.28352900	1.06945500	-1.37458000
H	-3.08638300	-0.51313100	-0.68186600
H	-4.14987900	-0.31924100	-2.11720800

8

E (SMD/M06-2X/6-31G(d)) = -3328.75188505

G (SMD/M06-2X/6-31G(d)) = -3328.592655

E (SMD/M06-2X/def2-TZVP//SMD/M06-2X/6-31G(d)) = -3329.82360198

C	2.44138700	-0.85388300	-0.08538300
C	3.27347900	-0.01046600	0.64877300
C	3.98043000	-0.57580600	1.75134500
C	3.41610900	1.37890200	0.36178000
C	4.78747600	0.21718100	2.53402900
H	3.86096000	-1.63732700	1.96284900
C	4.21717700	2.16099300	1.15805100
H	2.88956700	1.82527600	-0.47900100
C	4.90144000	1.58109400	2.23737800
H	5.32709800	-0.20429600	3.37772000
H	4.32084800	3.22386500	0.95574700
H	5.53612800	2.20886300	2.86026300
C	1.59511300	-0.49486000	-1.19410300
H	1.37174800	-1.32712000	-1.86994600
H	1.80680000	0.44241200	-1.71424200
H	2.40596500	-1.89770400	0.23253400
Au	-0.32985200	-0.16229700	-0.38397100
O	-2.40702700	0.17919000	0.19870200
S	-3.37983800	0.34271800	-0.96788500
O	-4.42552700	1.30959900	-0.67914600
O	-2.68201400	0.40397100	-2.25065300
C	-4.24205800	-1.28106300	-0.96818100
F	-4.91800600	-1.43624200	0.15815200
F	-5.08450900	-1.31310800	-1.99059500
F	-3.36755400	-2.26785500	-1.09418200
O	-0.65530900	-4.32779800	0.98569900
S	-0.35050300	-2.92661800	1.19612300
O	0.89486100	-2.53412800	1.83982000
O	-0.59626100	-2.18306500	-0.14268400
C	-1.67856700	-2.27594400	2.29562900
F	-1.66496700	-2.98793700	3.40885600
F	-1.44110500	-1.00632900	2.58473200
F	-2.84974100	-2.39380600	1.70436000
O	0.07126100	2.11536300	1.69749600
S	0.15641700	2.80844700	0.42084600
O	1.27008900	3.70009900	0.14577400
O	-0.05280200	1.83938300	-0.77605300
C	-1.34757800	3.85819400	0.27219000
F	-1.28586400	4.79375500	1.20457400
F	-1.37252600	4.42032900	-0.92217500
F	-2.42766500	3.12608000	0.44631200

9

E (SMD/M06-2X/6-31G(d)) = -3364.90533692

G (SMD/M06-2X/6-31G(d)) = -3364.758629

E (SMD/M06-2X/def2-TZVP//SMD/M06-2X/6-31G(d)) = -3366.00350736

C	1.69690000	2.34362200	-4.94918100

H	1.98495300	1.99394900	-5.95085100
H	2.38796900	3.12445800	-4.61882200
H	0.68255400	2.75903500	-5.03808900
C	1.71186700	1.19469600	-3.99538100
O	2.45272200	1.11283700	-3.04017000
C	0.72192100	0.06933700	-4.28500700
C	-0.64861600	0.31165800	-3.77189700
C	-1.72318300	-0.64608400	-4.08486100
H	-2.25583800	-0.26685500	-4.97240100
H	-1.31435900	-1.62958900	-4.33486200
H	-2.44633600	-0.72899800	-3.26402500
O	-0.82190200	1.35415700	-3.09409500
H	0.62683000	-0.14761200	-5.36179500
Au	-2.65025500	1.86221400	-2.31148900
O	-4.39495300	2.49927300	-1.49786700
S	-5.63467300	1.55989800	-1.70942700
O	-6.79391100	2.36349100	-2.03117300
O	-5.27149100	0.39562000	-2.50060300
C	-5.86300800	0.96426100	0.01973000
F	-6.09440100	1.99033000	0.81295700
F	-6.89195900	0.13782900	0.03649900
F	-4.77192800	0.33010400	0.40793400
O	-0.67850500	1.21306200	1.48262000
S	-1.14775100	2.04864700	0.39818700
O	-0.24052600	2.85500300	-0.39498800
O	-2.07627000	1.16046900	-0.49932300
C	-2.36918300	3.22264900	1.13642600
F	-1.77949300	3.85685700	2.13079000
F	-2.75425700	4.09550100	0.21933000
F	-3.41469700	2.55459100	1.58238900
O	-1.51959700	3.93696700	-4.64644700
S	-2.92626300	3.65379900	-4.86449700
O	-3.49585900	3.64182800	-6.19500900
O	-3.37368700	2.33271000	-4.15626100
C	-3.86681800	4.91093200	-3.89417200
F	-3.72566200	6.08059500	-4.48484300
F	-5.13891400	4.57235500	-3.84942700
F	-3.37644300	4.97003900	-2.66457800
H	1.06254800	-0.86377800	-3.80711000

10

E (SMD/M06-2X/6-31G(d)) = -3674.37747302
G (SMD/M06-2X/6-31G(d)) = -3674.104105
E (SMD/M06-2X/def2-TZVP//SMD/M06-2X/6-31G(d)) = -3675.58318641

C	0.92367000	1.85545500	-5.59839800
H	1.80459000	1.43003500	-6.10226300

H	0.92695500	2.94353900	-5.70824600
H	0.04116400	1.42506700	-6.09439600
C	0.95207300	1.46500100	-4.15622000
O	1.11493500	2.25840700	-3.25148200
C	0.72630700	-0.02431400	-3.89447200
C	-0.77364100	-0.25644700	-3.76417200
C	-1.33139700	-1.53276300	-4.27429700
H	-1.39212300	-1.45386500	-5.37113900
H	-0.66304900	-2.37627500	-4.05728200
H	-2.33250900	-1.72092800	-3.87196600
O	-1.46937100	0.62326600	-3.25539000
H	1.06003700	-0.59652900	-4.77379800
C	1.46146300	-0.64911100	-2.68318000
C	2.95423900	-0.86842000	-2.90399900
C	3.50848000	-2.08508500	-2.48537800
C	3.78799400	0.11223400	-3.45482000
C	4.86230900	-2.33671300	-2.66233400
H	2.86289600	-2.83604500	-2.02881200
C	5.14241400	-0.14550400	-3.63318300
H	3.39027200	1.08326100	-3.74671200
C	5.68084700	-1.36844200	-3.24079300
H	5.28021600	-3.29136800	-2.34842200
H	5.78107300	0.61708000	-4.07480100
H	6.74256800	-1.56480500	-3.37905000
C	1.51691000	0.08675100	-1.37379200
H	1.92961500	1.09797100	-1.40572200
H	1.89068700	-0.52474400	-0.54333700
H	1.02694400	-1.64038400	-2.50929200
Au	-0.44149300	0.51084300	-0.60216600
O	-2.82824900	1.07099500	-0.22539200
S	-4.11561000	0.90822400	-0.90076400
O	-5.14714500	1.89797500	-0.71616700
O	-3.93239300	0.60124900	-2.42361900
C	-4.81118100	-0.70428800	-0.33379900
F	-4.88824600	-0.66965900	0.98171200
F	-6.01059500	-0.85245900	-0.85735400
F	-4.02690200	-1.68857800	-0.71720400
O	-1.79614100	-3.68050100	-0.84124200
S	-0.81553100	-2.67623100	-0.47610400
O	0.60451600	-2.98233200	-0.57988200
O	-1.18652800	-1.34740500	-1.16112100
C	-1.09746900	-2.30619000	1.31512800
F	-1.15195700	-3.44779200	1.97344200
F	-0.09929000	-1.57352400	1.79245600
F	-2.23406200	-1.64578100	1.47059200
O	1.67756200	1.53147800	1.68933400
S	0.81115600	2.63617700	1.30614400
O	1.28827200	4.00316400	1.39302000

O	0.13404500	2.41779600	-0.06287200
C	-0.63273600	2.54516400	2.44546500
F	-0.22263600	2.80420200	3.67433200
F	-1.54813000	3.42582700	2.08479300
F	-1.16251300	1.32663300	2.41038100
H	-2.96450900	0.52534100	-2.73879600

TS_{10-11}

E (SMD/M06-2X/6-31G(d)) = -3674.37342622

G (SMD/M06-2X/6-31G(d)) = -3674.098968

E (SMD/M06-2X/def2-TZVP//SMD/M06-2X/6-31G(d)) = -3675.58099118

C	1.19831800	1.79941900	-5.62709900
H	2.08347200	1.26745700	-6.00588600
H	1.32811600	2.87751700	-5.75718300
H	0.34221200	1.44740400	-6.22091100
C	0.99385200	1.46224100	-4.18842000
O	1.04211300	2.27586900	-3.29037200
C	0.68807800	-0.01678500	-3.90314900
C	-0.79211400	-0.22170900	-3.76460700
C	-1.40672600	-1.48494200	-4.19921100
H	-1.52067000	-1.41367100	-5.29420400
H	-0.74002500	-2.33558900	-4.01042400
H	-2.39242900	-1.64824900	-3.75288200
O	-1.46020800	0.72241500	-3.27974600
H	-2.54257200	0.54675600	-2.94234700
H	1.00688200	-0.63118700	-4.75876100
C	1.42015400	-0.62832900	-2.66457400
C	2.90102300	-0.86333700	-2.92662600
C	3.41794600	-2.14343800	-2.70020000
C	3.76192100	0.16177400	-3.33593300
C	4.76532700	-2.40461500	-2.91616800
H	2.75237600	-2.93654300	-2.35935500
C	5.10970400	-0.10451700	-3.55300400
H	3.39290400	1.17695900	-3.47794200
C	5.61299800	-1.38605200	-3.34622000
H	5.15447500	-3.40716800	-2.74801900
H	5.77012000	0.69725900	-3.87784600
H	6.66906300	-1.59005700	-3.51367600
C	1.43950200	0.13006200	-1.35834100
H	1.84921900	1.14247400	-1.40329700
H	1.85794000	-0.46907500	-0.53986200
H	0.97906900	-1.61594500	-2.48821000
Au	-0.50054500	0.50701700	-0.60143200
O	-2.78295600	1.00278000	-0.18824900
S	-3.99869100	0.81490300	-1.01341000
O	-5.00623900	1.84960700	-0.90100600

O	-3.69111200	0.40534000	-2.43701200
C	-4.77300200	-0.72492300	-0.36802400
F	-4.92249700	-0.60675900	0.93989700
F	-5.95404900	-0.88220000	-0.93965900
F	-4.01199400	-1.76896100	-0.63544100
O	-1.64636400	-3.73877200	-0.70920400
S	-0.71756100	-2.67610700	-0.37647400
O	0.71611000	-2.90980000	-0.47408300
O	-1.15897900	-1.38977000	-1.10959100
C	-1.02005300	-2.26090200	1.40052000
F	-0.96714600	-3.37857800	2.09861700
F	-0.08614600	-1.42484300	1.83832500
F	-2.20807700	-1.69988600	1.53992500
O	1.59981600	1.71067300	1.65226800
S	0.65077900	2.73463100	1.24370700
O	1.01191700	4.13769500	1.30766100
O	0.01522200	2.44337200	-0.13378900
C	-0.79621900	2.54349500	2.37003300
F	-0.44114600	2.93130000	3.58243200
F	-1.80274600	3.28113800	1.93992100
F	-1.17252900	1.27055400	2.41330000

11

E (SMD/M06-2X/6-31G(d)) = -2712.56429374
G (SMD/M06-2X/6-31G(d)) = -2712.324882
E (SMD/M06-2X/def2-TZVP//SMD/M06-2X/6-31G(d)) = -2713.45284291

C	0.21123100	1.42159800	-5.63239600
H	1.02647100	1.05356000	-6.27366900
H	0.14566100	2.50934500	-5.72870700
H	-0.71339500	0.95233900	-5.99389200
C	0.51440000	1.03631300	-4.21767800
O	0.91790800	1.83602700	-3.39516500
C	0.28483900	-0.43639500	-3.89961400
C	-1.23418700	-0.63696400	-3.75268700
C	-1.78044500	-1.96421000	-4.16507100
H	-1.80317300	-1.99586300	-5.26525800
H	-1.13961900	-2.79380900	-3.83918100
H	-2.79616200	-2.09664200	-3.78044700
O	-1.93231400	0.27306400	-3.34605900
H	0.60030900	-1.02960600	-4.77363300
C	1.00989700	-1.03876600	-2.68385400
C	2.49897600	-1.33140000	-2.87168900
C	3.02530000	-2.47668800	-2.25537700
C	3.35144700	-0.48017200	-3.58751600
C	4.36688900	-2.79659700	-2.40234000
H	2.36425800	-3.11926500	-1.67241200
C	4.69295700	-0.80843500	-3.73552100

H	2.97281200	0.43497300	-4.03910000
C	5.20167000	-1.96398200	-3.14617700
H	4.76325300	-3.69654900	-1.93655500
H	5.34650200	-0.15385400	-4.30865100
H	6.25497800	-2.21305000	-3.26134700
C	1.19799500	-0.23773400	-1.44501300
H	1.62638800	0.76017300	-1.55785500
H	1.51766000	-0.80443100	-0.56425400
H	0.52984200	-1.99427400	-2.43793900
Au	-0.72796000	0.40052600	-0.53505500
O	-1.83387600	-3.86104700	-0.74658500
S	-1.30030700	-2.68154000	-0.08958300
O	0.06881700	-2.69055600	0.41033400
O	-1.62012600	-1.43649800	-0.93324500
C	-2.36110500	-2.39930300	1.38546800
F	-2.22287900	-3.41724400	2.21595200
F	-1.98924100	-1.28021400	1.99584500
F	-3.62475500	-2.29293400	1.01609800
O	-1.91025700	2.74638000	1.00195600
S	-0.66762100	3.29701600	0.47061000
O	0.11422200	4.22722000	1.26293000
O	0.22824600	2.19719500	-0.12313300
C	-1.14723700	4.21531900	-1.04844700
F	-1.92294800	5.23136800	-0.70754700
F	-0.06804700	4.66771900	-1.65976700
F	-1.81357200	3.41100500	-1.86437800

TS_{11-12}

E (SMD/M06-2X/6-31G(d)) = -2712.56422374

G (SMD/M06-2X/6-31G(d)) = -2712.324005

E (SMD/M06-2X/def2-TZVP//SMD/M06-2X/6-31G(d)) = -2713.45110518

C	0.76252800	1.86057200	-5.30743600
H	1.66489500	1.54760600	-5.85477500
H	0.72001700	2.95345400	-5.28526500
H	-0.09820800	1.45877100	-5.85756900
C	0.83928000	1.31100400	-3.91760300
O	1.10849100	2.00349700	-2.95481300
C	0.56370300	-0.18413400	-3.80579300
C	-0.96960300	-0.36470000	-3.86567000
C	-1.46925400	-1.68531000	-4.35718800
H	-1.35020900	-1.70621200	-5.45105600
H	-0.89599600	-2.53057900	-3.95805000
H	-2.52905200	-1.80243300	-4.11380200
O	-1.70581400	0.56017900	-3.58504700
H	0.96512200	-0.68073500	-4.70496500
C	1.14598100	-0.93553000	-2.60271900
C	2.65815700	-1.20567900	-2.60755400

C	3.58477000	-0.28445900	-3.12749400
C	3.11755700	-2.41838700	-2.06042000
C	4.93246600	-0.60725600	-3.16034700
H	3.25444300	0.67828800	-3.51231400
C	4.46580500	-2.73071700	-2.09331800
H	2.39688700	-3.11304800	-1.62859000
C	5.37294900	-1.82646100	-2.64637700
H	5.64560100	0.09896200	-3.57954700
H	4.81386400	-3.67759400	-1.68673300
H	6.43346500	-2.06990000	-2.66843300
C	1.36036400	-0.29374400	-1.30529500
H	1.77241700	0.71366800	-1.26103800
H	1.49474900	-0.93565100	-0.43257200
H	0.63808300	-1.89810900	-2.48236800
Au	-0.75890600	0.44792500	-0.43205400
O	-3.00278200	-3.29662000	-1.05776300
S	-2.75787600	-1.97528800	-0.50517100
O	-2.75715400	-1.77973500	0.93781800
O	-1.54545900	-1.33857100	-1.19348700
C	-4.13133200	-0.91982000	-1.12733400
F	-5.26196200	-1.31273900	-0.56428800
F	-3.90787300	0.35191900	-0.81225300
F	-4.23533000	-1.02931700	-2.44064000
O	-2.00657000	2.77686400	1.26698200
S	-0.71050600	3.27253300	0.81792200
O	0.03974900	4.19243400	1.65504600
O	0.19888400	2.15096500	0.30322600
C	-1.06637900	4.20209200	-0.72851600
F	-1.80982100	5.25666400	-0.42968600
F	0.06055000	4.60316300	-1.28986400
F	-1.73064600	3.43225700	-1.58080600

12
E (SMD/M06-2X/6-31G(d)) = -654.185416774
G (SMD/M06-2X/6-31G(d)) = -653.979086
E (SMD/M06-2X/def2-TZVP//SMD/M06-2X/6-31G(d)) = -654.432327261

C	0.13412100	1.20284200	-5.73139500
H	0.80091700	0.69439200	-6.44230800
H	0.18233300	2.28335400	-5.89381400
H	-0.88306000	0.84005200	-5.93809100
C	0.54904600	0.86799900	-4.33391200
O	1.00236400	1.69364300	-3.56585200
C	0.31662800	-0.58537100	-3.93828700
C	-1.19993200	-0.67953300	-3.58284700
C	-1.99219700	-1.69013400	-4.33891400
H	-1.93601500	-1.48292800	-5.41676500
H	-1.54951800	-2.68613700	-4.19109200

H	-3.03495400	-1.69087000	-4.00904500
O	-1.64018800	0.03679300	-2.71314500
H	0.51555700	-1.23342400	-4.80297900
C	0.99869300	-1.15076400	-2.71676300
C	2.62420600	-1.12741200	-2.51645900
C	3.15373000	-2.36470600	-2.04994100
C	3.43748200	-0.28461500	-3.32444600
C	4.43011200	-2.74857200	-2.39130400
H	2.52547300	-2.99813000	-1.42382100
C	4.71385300	-0.67989700	-3.65863800
H	3.05504600	0.67852300	-3.65163400
C	5.20379400	-1.90642600	-3.19745700
H	4.83492900	-3.69346000	-2.03902900
H	5.34256100	-0.03717300	-4.26908800
H	6.21375800	-2.20996100	-3.46748800
C	1.50864000	-0.43127000	-1.59532800
H	1.56319400	0.65239300	-1.64304000
H	1.47540800	-0.88477100	-0.60733500
H	0.67636800	-2.17573300	-2.51884100

13
E (SMD/M06-2X/6-31G(d)) = -1271.21587130
G (SMD/M06-2X/6-31G(d)) = -1271.080256
E (SMD/M06-2X/def2-TZVP//SMD/M06-2X/6-31G(d)) = -1271.65199430

C	2.83708900	-2.25380200	-4.50192900
C	1.99496000	-1.15487100	-5.09731300
C	2.94657100	-3.49025600	-5.34307800
C	2.53570400	-4.71900000	-4.82839600
C	3.47994900	-3.42566000	-6.63357400
C	2.65048100	-5.87472600	-5.59588700
H	2.13444400	-4.76896500	-3.81564600
C	3.60228500	-4.58059900	-7.39629000
H	3.82355700	-2.47007600	-7.03156200
C	3.18647500	-5.80650500	-6.87830700
H	2.32957100	-6.83136000	-5.18698700
H	4.02542600	-4.52651900	-8.39806200
H	3.28429500	-6.71102800	-7.47667500
H	0.97077400	-1.52366100	-5.23013800
H	1.97052200	-0.28192900	-4.43510600
H	2.37934400	-0.84651300	-6.07739200
H	2.48828500	-2.50633600	-3.49311600
O	4.18241700	-1.63324600	-4.34538200
S	5.23234300	-2.23980700	-3.32536100
O	6.06952000	-1.14613700	-2.88690900
O	4.60241800	-3.15189200	-2.39322800
C	6.31285300	-3.25288300	-4.43646600
F	7.43640900	-3.48508100	-3.78069600

F	6.57022500	-2.57124800	-5.53666100
F	5.73692500	-4.39785600	-4.73824400

TS$_{2-13}$
E (SMD/M06-2X/6-31G(d)) = -1271.17419611
G (SMD/M06-2X/6-31G(d)) = -1271.04877
E (SMD/M06-2X/def2-TZVP//SMD/M06-2X/6-31G(d)) = -1271.61806096

C	3.39857900	-2.74578300	-5.31920800
C	2.90967900	-4.01452600	-5.77633200
C	2.50507200	-4.95486400	-4.81125700
C	2.82618000	-4.34903800	-7.14141100
C	2.02379900	-6.19573000	-5.19687900
H	2.56654100	-4.68887400	-3.75621900
C	2.34456600	-5.58919200	-7.52232000
H	3.14582100	-3.63523500	-7.89865400
C	1.94382500	-6.51104800	-6.55215000
H	1.70838500	-6.91757400	-4.44667600
H	2.28195400	-5.84805400	-8.57720600
H	1.56799300	-7.48583200	-6.85857000
H	3.49713800	-2.65087500	-4.23462700
C	3.67856700	-1.61991800	-6.07432600
H	4.33440900	-0.87356700	-5.61795700
H	3.74362800	-1.69964100	-7.16224500
C	-0.45692700	-1.62177200	-6.67088500
F	-1.50371400	-2.35320500	-6.31866600
F	-0.85031300	-0.67448100	-7.50845000
F	0.42945700	-2.39884300	-7.28382900
S	0.31070900	-0.87610100	-5.18413600
O	-0.68171900	0.04624100	-4.65867500
O	0.72321000	-2.01583400	-4.36895600
O	1.49859200	-0.15738600	-5.80315800
H	2.54847800	-0.95637800	-5.89541800

TS$_{13-14}$
E (SMD/M06-2X/6-31G(d)) = -1580.58866425
G (SMD/M06-2X/6-31G(d)) = -1580.33513
E (SMD/M06-2X/def2-TZVP//SMD/M06-2X/6-31G(d)) = -1581.14740764

C	3.80430800	-3.83606300	-4.65220000
C	3.16780800	-1.24601100	-5.45657400
C	3.17817400	-4.84542500	-5.47464500
C	2.14094200	-5.61319800	-4.91732400
C	3.55470700	-5.08040300	-6.81014700
C	1.49230300	-6.58079700	-5.67063300
H	1.84949400	-5.43448100	-3.88227300
C	2.90513200	-6.04815300	-7.55971800
H	4.36813200	-4.50901900	-7.25517000

C	1.87315900	-6.79826200	-6.99330400
H	0.68866400	-7.16672500	-5.22900700
H	3.20395400	-6.22567000	-8.59105700
H	1.36752800	-7.55832500	-7.58641000
H	3.47511200	-3.81367000	-3.60981500
H	2.92357200	-1.19847700	-4.39695500
C	4.37644200	-0.06660500	-7.28349400
C	4.12778000	-0.27468300	-5.91489200
C	4.80884400	0.50794500	-4.96316400
C	5.71993900	1.46790200	-5.36751600
C	5.96162100	1.66035000	-6.72896200
C	5.28685100	0.89861600	-7.68382000
H	3.84985500	-0.65869200	-8.03031200
H	4.60366500	0.34802900	-3.90421200
H	6.24105300	2.07285200	-4.62851400
H	6.67891300	2.41415800	-7.04898900
H	5.47439300	1.06265300	-8.74289000
C	4.69291600	-2.88545900	-5.05340000
H	5.20402400	-2.28136400	-4.30703400
C	2.18181500	-1.87687000	-6.34864400
H	2.63768000	-2.34198900	-7.23296700
H	1.56656700	-2.61528900	-5.82655600
H	1.52204800	-1.06534800	-6.70761600
H	5.12381700	-2.88309100	-6.05491900
C	1.48268100	2.99694500	-5.82170900
F	0.85838800	3.79504600	-6.68590000
F	1.58793700	3.64109200	-4.66072500
F	2.71074200	2.76786000	-6.28281100
S	0.56106000	1.42788600	-5.61325200
O	-0.75498500	1.87215200	-5.14320400
O	0.59194100	0.85495100	-6.97039100
O	1.37029000	0.69700100	-4.62053600

14

E (SMD/M06-2X/6-31G(d)) = -1580.64834622
G (SMD/M06-2X/6-31G(d)) = -1580.387117
E (SMD/M06-2X/def2-TZVP//SMD/M06-2X/6-31G(d)) = -1581.19784741

C	3.85082300	-3.99277100	-5.87941300
C	3.96411800	-1.56006600	-5.05945600
C	3.89999500	-4.93376200	-7.04773500
C	2.74041500	-5.60200300	-7.43962900
C	5.08473900	-5.13021700	-7.76228900
C	2.75708900	-6.44731500	-8.54544900
H	1.82095600	-5.46041600	-6.87006300
C	5.10320800	-5.98418700	-8.85901400
H	5.99702800	-4.62214900	-7.44853200
C	3.93876700	-6.63987800	-9.25500100

H	1.84757000	-6.96535900	-8.84523900
H	6.03074900	-6.13975600	-9.40762200
H	3.95560900	-7.30754000	-10.11505600
H	2.93305500	-4.16454700	-5.30210700
H	4.82377700	-1.80245800	-4.41305100
C	3.14993200	0.53221900	-6.24161600
C	4.16813000	-0.14974900	-5.56728900
C	5.39739600	0.49117500	-5.39869200
C	5.60697500	1.77996100	-5.88318100
C	4.58434400	2.44952200	-6.54874800
C	3.35425800	1.81990700	-6.72712100
H	2.18184800	0.05128800	-6.38926100
H	6.20084100	-0.03056400	-4.87601600
H	6.57262000	2.26264400	-5.73721000
H	4.74372700	3.45831500	-6.92698800
H	2.54791000	2.33555600	-7.24742700
C	3.98715000	-2.53078100	-6.24884200
H	4.91493300	-2.39307900	-6.82616000
C	2.69130500	-1.69809800	-4.22806600
H	1.78990600	-1.61161600	-4.85193700
H	2.64392000	-2.66698000	-3.71311200
H	2.64288700	-0.91759500	-3.45777300
H	3.15459600	-2.30075400	-6.93324900
C	5.39268400	-4.59309700	-2.47173800
F	6.54577400	-3.97702900	-2.64379300
F	4.44989300	-3.71045300	-2.19709500
F	5.48858800	-5.46674700	-1.48718300
S	4.94855600	-5.50820300	-4.00600900
O	4.99346300	-4.25566400	-4.97864600
O	6.05107100	-6.39731000	-4.29698700
O	3.59554500	-6.00029400	-3.84394800

15

E (SMD/M06-2X/6-31G(d)) = -618.8305171

G (SMD/M06-2X/6-31G(d)) = -618.60346

E (SMD/M06-2X/def2-TZVP//SMD/M06-2X/6-31G(d)) = -619.063825949

C	3.33122500	-3.43179700	-4.53542900
C	3.11914000	-1.38088100	-5.97106200
C	3.82437700	-4.75358800	-4.13840900
C	3.35516400	-5.31970700	-2.94503700
C	4.74872800	-5.48784700	-4.89737200
C	3.78974600	-6.57097800	-2.52107000
H	2.63648600	-4.76110900	-2.34374700
C	5.18286000	-6.73714100	-4.47582200
H	5.13366100	-5.07718000	-5.83036800
C	4.70647900	-7.28650500	-3.28547200

H	3.40982700	-6.98799800	-1.58927600
H	5.90059800	-7.28944300	-5.08120700
H	5.04925600	-8.26682900	-2.95793200
H	2.60376700	-2.98726600	-3.84813700
H	2.39557200	-1.10048500	-5.18869500
C	5.25936500	-0.37952800	-6.90691400
C	4.21947900	-0.33669600	-5.97202700
C	4.22096600	0.68722600	-5.02309500
C	5.22888300	1.64823100	-5.00693200
C	6.25807100	1.59562400	-5.94220000
C	6.27012500	0.57633900	-6.89217700
H	5.28162800	-1.17120300	-7.65732500
H	3.41747500	0.73037000	-4.28629500
H	5.20902900	2.44063700	-4.25959000
H	7.04854100	2.34474700	-5.93206600
H	7.07204000	0.52580000	-7.62792800
C	3.67361000	-2.73325500	-5.62462300
C	2.38456700	-1.43222500	-7.31231300
H	3.05257400	-1.76140500	-8.12051100
H	1.54617500	-2.13977100	-7.26602400
H	1.98943800	-0.44401100	-7.58331000
H	4.39896500	-3.13888600	-6.33809900

$TS_{13\text{-}16}$
E (SMD/M06-2X/6-31G(d)) = -1616.78074596
G (SMD/M06-2X/6-31G(d)) = -1616.535857
E (SMD/M06-2X/def2-TZVP//SMD/M06-2X/6-31G(d)) = -1617.36406279

C	3.24575900	-3.51696000	-4.11546800
C	4.15802700	-2.72541100	-3.27543600
C	2.90373200	-3.15196200	-5.46382500
C	2.23802200	-4.09400700	-6.27160400
C	3.23619000	-1.89450200	-5.99926400
C	1.90101700	-3.78229800	-7.57738400
H	1.99723000	-5.07206800	-5.85376300
C	2.90012900	-1.58879700	-7.30936900
H	3.75918700	-1.16050500	-5.38853800
C	2.22929600	-2.52687900	-8.09419500
H	1.38979300	-4.51325800	-8.20019600
H	3.16189000	-0.61798000	-7.72457900
H	1.96515400	-2.28074500	-9.12123500
H	3.11731800	-4.56947600	-3.86750500
H	5.14019900	-2.74362300	-3.78342700
H	3.85944700	-1.67161700	-3.19070600
H	4.28701000	-3.15629200	-2.27851800
C	1.19436800	-3.17118100	-3.07753300
H	0.71369200	-3.96981000	-3.63916000
C	1.62851200	-3.46668000	-1.78546500

C	0.93643900	-1.78835100	-3.45895300
C	1.73038300	-4.84366800	-1.26105200
H	1.32077200	-5.57688300	-1.96108700
H	2.78708200	-5.07928200	-1.06604400
H	1.20674600	-4.91605600	-0.29931600
C	0.09650500	-1.53465200	-4.66530500
H	0.27878700	-0.52768400	-5.05425500
H	0.25809900	-2.28738500	-5.44682000
H	-0.96018900	-1.60438200	-4.36631900
O	2.03654600	-2.53513600	-0.96630500
O	1.38821100	-0.84805900	-2.78553900
H	1.91809100	-1.65228800	-1.43699100
C	6.30367900	-4.83833700	-7.21652700
F	7.40807100	-4.40554200	-7.82121800
F	6.08205000	-6.09375800	-7.60166000
F	5.28382500	-4.08960500	-7.63195100
S	6.48833400	-4.74157100	-5.39678200
O	7.65542500	-5.58903100	-5.13411400
O	6.68263200	-3.29905800	-5.16863800
O	5.20275600	-5.27091300	-4.90304600

16
E (SMD/M06-2X/6-31G(d)) = -1616.79935675
G (SMD/M06-2X/6-31G(d)) = -1616.553285
E (SMD/M06-2X/def2-TZVP//SMD/M06-2X/6-31G(d)) = -1617.38388242

C	3.18591300	-3.32039500	-4.09043100
C	4.27239200	-2.50894300	-3.41177700
C	2.96821000	-2.99631700	-5.54795400
C	2.40910400	-3.97125000	-6.38003900
C	3.26722000	-1.74271000	-6.08381000
C	2.13994700	-3.69706200	-7.71544900
H	2.19150400	-4.95907100	-5.97002100
C	3.00291700	-1.46728600	-7.42372700
H	3.70895600	-0.96837500	-5.45735100
C	2.43463400	-2.43951000	-8.24038200
H	1.70971500	-4.46874800	-8.35205800
H	3.24495100	-0.48617100	-7.82936900
H	2.22789700	-2.22228300	-9.28715400
H	3.46232600	-4.38151900	-4.03097500
H	5.18645000	-2.57852400	-4.01584500
H	4.01463400	-1.44766400	-3.29295600
H	4.51635900	-2.91139800	-2.41924700
C	1.77022300	-3.22344800	-3.32346800
H	1.13595600	-4.00229600	-3.76553900
C	2.03495000	-3.55602500	-1.90762200
C	1.15983400	-1.85127800	-3.51371100
C	2.20457500	-4.93998400	-1.46657500

H	1.44133000	-5.58746000	-1.91303700
H	3.17774400	-5.29423000	-1.84785500
H	2.20908000	-5.01165900	-0.37583000
C	0.18520000	-1.65558800	-4.61514200
H	0.12426400	-0.59714200	-4.88615300
H	0.40868400	-2.27537600	-5.49032200
H	-0.79792100	-1.97435400	-4.23379500
O	2.20198800	-2.63793600	-1.04403200
O	1.45732500	-0.94080900	-2.73992900
H	2.04440300	-1.73100500	-1.49784300
C	6.38951400	-5.14217400	-7.25234200
F	7.53864100	-4.83515500	-7.85334600
F	6.06192000	-6.38709500	-7.59859100
F	5.44947400	-4.32189000	-7.71849500
S	6.55292400	-4.99409800	-5.43344000
O	7.65132300	-5.91829500	-5.12536800
O	6.85878100	-3.56586500	-5.25365100
O	5.22731400	-5.41585400	-4.94824000

HOTF

E (SMD/M06-2X/6-31G(d)) = -961.78310666
G (SMD/M06-2X/6-31G(d)) = -961.776143
E (SMD/M06-2X/def2-TZVP//SMD/M06-2X/6-31G(d)) = -962.113177064

C	2.95108300	-3.54024800	-5.48928300
F	3.12864900	-4.47173800	-4.57128200
F	1.71132200	-3.57042200	-5.93076500
F	3.79331400	-3.72161400	-6.48714000
S	3.29838900	-1.89356500	-4.74267900
O	2.19300600	-1.89048400	-3.58964300
H	2.56774000	-2.24753900	-2.75365000
O	4.62267100	-1.96130200	-4.16653800
O	2.90645600	-0.89826000	-5.70810800

OTF

E (SMD/M06-2X/6-31G(d)) = -961.354424
G (SMD/M06-2X/6-31G(d)) = -961.350965979
E (SMD/M06-2X/def2-TZVP//SMD/M06-2X/6-31G(d)) = -961.686133454

C	2.94947400	-3.50326400	-5.46093600
F	3.14469200	-4.50247400	-4.59912100
F	1.70470700	-3.60373200	-5.92946700
F	3.78723000	-3.67579800	-6.48461100
S	3.21952800	-1.87902100	-4.65810900
O	2.20802400	-1.87653100	-3.59153900
O	4.61446200	-1.97275900	-4.20390100
O	2.97677200	-0.93405300	-5.75775300

water-11

E (SMD/M06-2X/6-31G(d)) = -2788.97843077

G (SMD/M06-2X/6-31G(d)) = -2788.710503

E (SMD/M06-2X/def2-TZVP//SMD/M06-2X/6-31G(d)) = -2789.90341642

C	0.43727400	1.42176500	-5.55022300
H	1.28445600	0.95582400	-6.07522800
H	0.46988000	2.50600600	-5.69172000
H	-0.47709700	1.00921100	-6.00140700
C	0.50949500	1.07213700	-4.09615100
O	0.72588600	1.89743400	-3.23032300
C	0.25460500	-0.39615400	-3.78274700
C	-1.26322700	-0.57988300	-3.62946300
C	-1.82931400	-1.84910900	-4.17168800
H	-1.80591700	-1.80336500	-5.27093300
H	-1.21208300	-2.71008500	-3.88019500
H	-2.86030000	-1.98971500	-3.83362300
O	-1.94586100	0.27927000	-3.09687100
H	0.55704200	-0.99579200	-4.65508500
C	0.99404000	-1.00698300	-2.56888600
C	2.46093700	-1.31455100	-2.84248100
C	2.98035700	-2.53702800	-2.40283300
C	3.31369600	-0.39909500	-3.46842700
C	4.31544700	-2.85589400	-2.61970400
H	2.32334800	-3.24313700	-1.89343900
C	4.64892300	-0.72081300	-3.68719400
H	2.94415300	0.57512300	-3.78689900
C	5.15213100	-1.94916800	-3.26634500
H	4.70304900	-3.81613200	-2.28383200
H	5.30009700	-0.00420200	-4.18468300
H	6.19815900	-2.19759400	-3.43745200
C	1.07923200	-0.21499800	-1.28627700
H	1.55795400	0.76321100	-1.37921200
H	1.46197100	-0.81242500	-0.44925000
H	0.51123400	-1.97010700	-2.35359000
Au	-0.80475800	0.32661300	-0.52113900
O	-1.86831200	-3.97339400	-0.61803900
S	-1.22899300	-2.79849000	-0.05276100
O	0.17795900	-2.84857700	0.31951000
O	-1.58016200	-1.57459200	-0.91528600
C	-2.13498500	-2.44336200	1.51014200
F	-2.00744700	-3.48362300	2.31637800
F	-1.62249600	-1.37051200	2.10139600
F	-3.41268100	-2.23384200	1.25377900
O	-1.88436600	3.09066300	1.08893800
S	-0.61273700	3.38703100	0.42508600
O	0.29678500	4.32629800	1.04997000
O	0.14065200	2.12974700	-0.01492400

C	-1.09415300	4.13355700	-1.18584500
F	-1.80708600	5.22292500	-0.94815100
F	-0.01986300	4.44919300	-1.87854900
F	-1.83269100	3.26917300	-1.86965400
O	-2.98505200	0.78058800	0.13367400
H	-3.51429800	0.86790300	-0.68045800
H	-2.94426000	1.67412000	0.53726700

water-TS$_{13\text{-}16}$

E (SMD/M06-2X/6-31G(d)) = -1693.1758712

G (SMD/M06-2X/6-31G(d)) = -1692.911923

E (SMD/M06-2X/def2-TZVP//SMD/M06-2X/6-31G(d)) = -1693.79965549

C	3.44679100	-3.18868300	-4.45590100
C	4.20822200	-2.38709200	-3.48351700
C	3.04625200	-2.72068000	-5.73563800
C	2.67139800	-3.66903200	-6.71411400
C	3.03092600	-1.34493400	-6.05730300
C	2.31415400	-3.25023200	-7.98540400
H	2.71652200	-4.73005000	-6.46749100
C	2.65493100	-0.93714300	-7.32235500
H	3.30400400	-0.60678500	-5.30541300
C	2.30231700	-1.88868600	-8.28556200
H	2.04936000	-3.98117300	-8.74591000
H	2.63625700	0.12137500	-7.57175700
H	2.01596000	-1.56032400	-9.28326500
H	3.37078300	-4.26460100	-4.28461600
H	5.21397500	-2.24969100	-3.91113000
H	3.78926600	-1.38622100	-3.32690500
H	4.32755800	-2.90369800	-2.52713400
C	1.29310200	-3.12832300	-3.04286600
H	1.19753700	-2.05381500	-2.88695000
C	0.58216600	-3.63294400	-4.11677900
C	1.81698500	-3.90426400	-1.92598200
C	-0.21815600	-2.73239000	-4.98669300
H	-0.15299200	-1.68605400	-4.67551600
H	0.11997400	-2.83216800	-6.02888100
H	-1.27007400	-3.04813900	-4.96550600
C	2.08077100	-3.13408800	-0.66252300
H	2.71142200	-3.72075300	0.01302800
H	2.54375000	-2.15952300	-0.86370500
H	1.12302500	-2.93343000	-0.16035100
O	0.53077700	-4.87676400	-4.51347400
O	2.00228000	-5.12040000	-1.96982700
H	1.18749600	-5.52394100	-4.10493800
C	6.21897600	-5.46789700	-7.38034700
F	7.42005500	-4.90108300	-7.43683600
F	6.26174400	-6.61714900	-8.04539900

F	5.34566200	-4.66451000	-7.98221900
S	5.73620300	-5.75330300	-5.63704300
O	6.76166000	-6.66324800	-5.12855500
O	5.73580500	-4.39759200	-5.05988400
O	4.38498200	-6.34092900	-5.78796700
O	2.26280000	-6.80003700	-4.06097300
H	3.04363500	-6.70548000	-4.64695400
H	2.57520600	-6.57709800	-3.16845300

water-Au(OTf)$_3$

E (SMD/M06-2X/6-31G(d)) = -3095.7

G (SMD/M06-2X/6-31G(d)) = -3095.6

E (SMD/M06-2X/def2-TZVP//SMD/M06-2X/6-31G(d)) = -3096.7

Au	-0.85825100	0.51153400	-0.13898800
O	-2.07221800	-3.55867700	-0.27948400
S	-1.65499500	-2.45931500	0.56599100
O	-0.30258000	-2.37437900	1.08463700
O	-2.07656300	-1.11687500	-0.13112500
C	-2.80066200	-2.48633100	2.01448100
F	-2.65466000	-3.65713800	2.60717600
F	-2.50242600	-1.51961100	2.85595200
F	-4.04011300	-2.34906400	1.58674000
O	-1.53362300	3.64350800	0.37263000
S	-0.27783300	3.57509700	-0.34681700
O	0.80906600	4.49665000	-0.10242600
O	0.33420500	2.12432200	-0.35724600
C	-0.72153100	3.75182700	-2.12823300
F	-1.13106500	4.98765600	-2.33404700
F	0.32847400	3.49110600	-2.88215400
F	-1.69818900	2.90436900	-2.41872400
O	-0.51035500	0.19632000	-2.18909900
H	0.44054500	-0.00341400	-2.34994900
H	-1.02400000	-0.57487400	-2.52272900
O	-1.30815300	0.74723500	1.80192300
S	-0.39098900	1.38181300	2.91723500
O	0.39321200	2.50583900	2.45665200
O	-1.23488300	1.46347500	4.09032900
C	0.81571800	0.02106000	3.22032800
F	1.67338300	0.43521100	4.13289400
F	0.18644800	-1.05091100	3.64666200
F	1.45920000	-0.24407300	2.09369700

water-HOTf

E (SMD/M06-2X/6-31G(d)) = -1038.18887045

G (SMD/M06-2X/6-31G(d)) = -1038.159987

E (SMD/M06-2X/def2-TZVP//SMD/M06-2X/6-31G(d)) = -1038.56096662

C	2.86981800	-3.48497300	-5.63420300
F	3.24977500	-4.30171100	-4.66230400
F	1.58794300	-3.66125700	-5.88571800
F	3.57832600	-3.72735400	-6.72087200
S	3.17267600	-1.75289900	-5.10470900
O	2.28912400	-1.70936000	-3.80952100
H	2.83416600	-2.03476500	-3.00143100
O	4.58212800	-1.67891200	-4.77096100
O	2.57653300	-0.89879700	-6.10466900
O	3.75289900	-2.56978600	-1.86305000
H	3.90518000	-3.52687200	-1.94685600
H	4.63375000	-2.16809300	-1.96154300

1 AgCl
E (SMD/M06-2X/6-31G(d)) = -607.218381736
G (SMD/M06-2X/6-31G(d)) = -607.242128
E (SMD/M06-2X/def2-TZVP//SMD/M06-2X/6-31G(d)) = -607.286629562

Cl	1.06396000	1.30523600	-1.87450500
Ag	-0.52749200	-0.24800900	-1.00138800

4 AgCl
E (SMD/M06-2X/6-31G(d)) = -2428.99477426
G (SMD/M06-2X/6-31G(d)) = -2429.042035
E (SMD/M06-2X/def2-TZVP//SMD/M06-2X/6-31G(d)) = -2429.26169822

Cl	3.34562400	3.10219100	-0.82371300
Cl	1.26190800	3.37763600	-4.61920400
Ag	2.94476200	1.85622600	-3.15227200
Ag	2.26560700	4.79743600	-2.58763900
Ag	5.31060800	3.83701200	-2.53730000
Cl	5.26677500	1.98702100	-4.47904500
Ag	3.77725700	4.09531700	-5.23052000
Cl	4.37360100	6.13004200	-3.56530300

6 AgCl
E (SMD/M06-2X/6-31G(d)) = -3643.54315578
G (SMD/M06-2X/6-31G(d)) = -3643.595846
E (SMD/M06-2X/def2-TZVP//SMD/M06-2X/6-31G(d)) = -3643.94894654

Cl	3.34632900	3.13558400	-1.07376900
Cl	0.20804400	2.16479300	-4.49020900
Ag	2.53973400	2.28469300	-3.58214100
Ag	1.94638100	4.89514100	-2.09806300
Ag	4.93692700	4.49335500	-2.69197100
Ag	0.92521600	4.47506900	-5.08586500
Cl	5.04836000	2.30061500	-4.39529200
Cl	0.80838900	6.66750700	-3.38249000

Ag	3.92299100	4.07550000	-5.68733400
Ag	3.31842800	6.68840100	-4.20077500
Cl	5.64667400	6.80620200	-3.28619600
Cl	2.51496100	5.83436700	-6.70340100

12 AgCl

E (SMD/M06-2X/6-31G(d)) = -7287.11823441

G (SMD/M06-2X/6-31G(d)) = -7287.200696

E (SMD/M06-2X/def2-TZVP//SMD/M06-2X/6-31G(d)) = -7287.92361869

Ag	1.05127000	3.06076500	-3.44399900
Cl	0.49511400	3.85042900	-1.12020000
Cl	1.97061800	2.43415600	-5.68899300
Ag	1.94855800	5.88827400	-2.78425700
Ag	-1.21399600	5.85672600	-2.55854800
Ag	0.79647500	5.09396900	-5.73832400
Cl	0.68934600	7.73600500	-4.16636800
Cl	-1.35841800	4.37733600	-4.57221900
Ag	3.11600700	7.91982100	-4.75708300
Cl	2.39290300	6.33094100	-7.25678700
Cl	2.96980100	10.03756200	-1.96186400
Cl	3.11986500	6.83429900	1.42113400
Cl	-1.07170400	7.45717000	-0.58361600
Ag	0.97881500	8.64992000	-1.46458200
Ag	1.12063200	6.04984000	0.16119000
Ag	4.71544500	5.77723900	-6.55571200
Cl	5.56008100	8.10265100	-5.12850300
Ag	4.88404400	8.52253800	-2.27290000
Ag	4.33559400	3.11458100	-5.38262500
Cl	4.05405800	4.47251100	-2.76908300
Ag	6.42712500	5.73916600	-3.81751500
Cl	6.59993900	4.14349100	-5.85932400
Cl	6.66091900	6.90178100	-1.53524300
Ag	4.20050000	6.09154300	-0.67950100

18 AgCl

E (SMD/M06-2X/6-31G(d)) = -10930.7137162

G (SMD/M06-2X/6-31G(d)) = -10930.824502

E (SMD/M06-2X/def2-TZVP//SMD/M06-2X/6-31G(d)) = -10931.915287

Cl	-0.03260000	-0.18271500	-2.89020400
Ag	1.21837700	-0.13890500	-0.76766700
Ag	-0.56913200	2.25244800	-3.58433400
Cl	2.48399100	0.38945900	1.29316000
Cl	-0.18301500	3.69108800	-1.25848500
Ag	1.47974500	2.63708400	0.37376700
Cl	4.14812100	-0.58686200	-2.05266700
Cl	3.46697100	3.26420600	-1.63991900

Cl	1.79377400	2.34485300	-5.35670600
Ag	2.42467900	1.18634900	-3.13566800
Ag	4.82152800	1.14249000	0.01874800
Ag	2.02046400	5.25726100	-2.53723800
Ag	-1.60769500	5.80596400	-2.47816400
Ag	6.02330300	3.26479400	-3.08002800
Ag	0.65393200	4.81832100	-5.42766700
Cl	5.81122400	2.25424000	-5.62961800
Cl	0.54259600	7.01994800	-3.79226800
Cl	-1.83023000	4.25253600	-4.59840400
Ag	5.22248700	0.53985600	-3.97232700
Ag	3.83489300	4.07867400	-5.13774700
Ag	3.28848000	7.41083700	-4.48086600
Cl	7.18727300	1.81820500	-1.20528900
Cl	5.61084400	5.74799200	-3.82975300
Cl	2.75193300	6.02547900	-6.58673600
Cl	6.62409200	6.48395900	0.09368400
Cl	3.36991400	9.13753300	-2.54256600
Cl	2.59225800	6.50253000	0.16208700
Cl	5.34027100	2.86415200	2.14276300
Cl	0.60357300	3.89375200	2.81063500
Cl	-1.13414500	7.41851600	-0.44098000
Ag	7.18828200	4.16993300	-0.46320400
Ag	4.72678900	7.05719400	-1.66784100
Ag	1.11781300	8.37050700	-1.41681600
Ag	4.44567200	4.79633000	0.62898200
Ag	3.01383600	3.39289300	2.80651900
Ag	0.08878400	5.59534500	0.93863200

References

1. X. Yao and Ch.-J. Li. *J. Am. Chem. Soc.*, **2004**,126, 6884-6885.
2. P. N. Liu, L. Dang, Q. W. Wang, Sh. L. Zhao, Fe. Xia, Y. J. Ren, X. Q. Gong, J. Q. Chen. *J. Org. Chem*. **2010**, 75, 5017–5030.
3. Z. S. Qureshi, K. M. Deshmukh, P. J. Tambade, K. P. Dhake, B. M. Bhanage. *Eur. J. Org. Chem.* **2010***,* 6233–6238.
4. K. Farshadfar, A. J. Tague, M. Talebi, B. F. Yates, C. J. T. Hyland, A. Ariafard. *ACS Catal.* **2022**, 12, 7918–7925
